# Biomimetic Apatite Nanoparticles and Microcrystalline Tyrosine as Biocompatible Vaccine Adjuvants: Performance in a Bluetongue Virus Sheep Model

**DOI:** 10.1021/acsami.5c10402

**Published:** 2025-07-08

**Authors:** Estela Pérez, Víctor Sebastián, Ana Rodríguez-Largo, Ricardo de Miguel, Álex Gómez, Matthias F. Kramer, Anke Graessel, Belén Parra-Torrejón, José Manuel Delgado-López, Sergio Utrilla-Trigo, Luis Jiménez-Cabello, Javier Ortego, Ignacio de Blas, Ramsés Reina, Marta Pérez, Lluís Luján

**Affiliations:** † Department of Animal Pathology, 16765 University of Zaragoza, 177 Miguel Servet Street, Zaragoza 50013, Spain; ‡ Institute of Nanoscience and Materials of Aragon (INMA), CSIC-University of Zaragoza, Mariano Esquillor Gómez Street I+D+i building, Zaragoza 50018, Spain; § Department of Chemical and Environmental Engineering, 16765 University of Zaragoza, María de Luna 3 Street, Zaragoza 50018, Spain; ∥ Advanced Microscopy Laboratory, University of Zaragoza, Mariano Esquillor Gómez Street I+D+i building, Zaragoza 50018, Spain; ⊥ Agri-Food Institute of Aragon (IA2), University of Zaragoza, 177 Miguel Servet Street, Zaragoza 50013, Spain; # Bencard Adjuvant Systems, Allergy Therapeutics PLC, Dominion Way Street, Worthing BN14 8SA, U.K.; g Department of Inorganic Chemistry, University of Granada, Fuentenueva avenue, Granada 18071, Spain; h Center of Research in Animal Health (CISA-INIA, CSIC), Algete-El Casar road, km. 8.1, Madrid 28130, Spain; i Institute of Agrobiotechnology, CSIC-Government of Navarra, Avenue of Pamplona, Mutilva 31192, Spain; j Department of Anatomy, Embryology and Genetics, University of Zaragoza, 177 Miguel Servet Street, Zaragoza 50013, Spain

**Keywords:** aluminum oxyhydroxide adjuvant, biomimetic apatite nanoparticles, microcrystalline tyrosine, sheep, vaccine, bluetongue virus, ovine model

## Abstract

Aluminum oxyhydroxide (AlOOH) is the most widely used vaccine adjuvant, but its known adverse effects prompt the finding of other, safer alternative adjuvants. This study compares in sheep the global performance and safety of vaccine prototypes against bluetongue virus (BTV) formulated with AlOOH, biomimetic apatite nanoparticles (ApNPs), and microcrystalline tyrosine (MCT). Five groups of 6 sheep were included in the study: control, BTV serotype 4 alone, and BTV-4 combined either with AlOOH, ApNPs, or MCT. Adjuvants were fully characterized. Group specific antibodies against BTV-4 were observed in all treatment groups, including BTV-4 alone. After booster inoculation, ApNPs or MCT groups responded immediately, and it was delayed for BTV-4 and AlOOH groups. Comparable neutralizing antibody responses were observed in all treatment groups but were earlier for BTV-4, ApNPs, and MCT groups when compared with the AlOOH group. No significant systemic alterations were observed during the study. The AlOOH group developed more pronounced local reactions that persisted throughout the 133-day study and were evident as post-mortem granulomas. ApNPs and MCT are biocompatible, safer, and viable alternative adjuvants for sheep vaccines. BTV alone might also be suitable for this purpose. This work is the first demonstrating the suitability of biocompatible alternative adjuvants in sheep vaccines.

## Graphical abstract

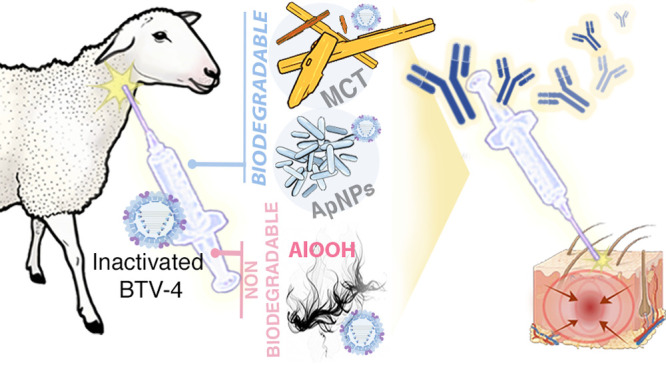

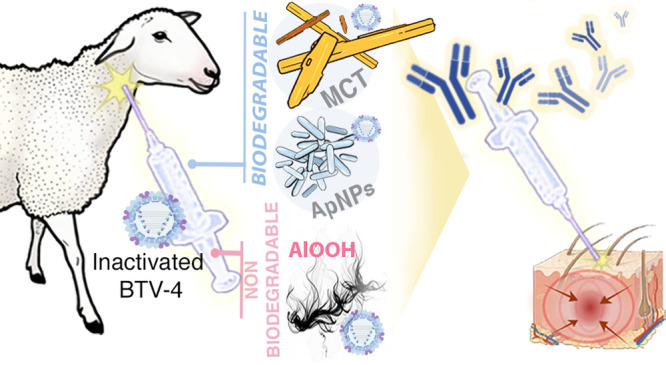

## 1 Introduction

Aluminum oxyhydroxide (AlOOH) is the most widely used vaccine adjuvant in human and veterinary medicine.^1^ It exhibits limited capacity to induce a cellular immune response, with its immunological effect predominantly characterized by a Th2 profile, which is more effective against extracellular pathogens.^2^ However, safety concerns have been raised regarding its adverse effects.^3^ Al, a nonbiodegradable metal, leads to long-term accumulation of AlOOH adjuvant nanoparticle aggregates within intracytoplasmic macrophage phagolysosomes at injection site (IS) granulomas.^4,5^ These are occasionally associated with conspicuous, Al-based, rod- or cigar-shaped hyaline structures ranging about 30–100 μm, known as crystalloid bodies.^5^ The nature of these bodies and their relationship to local inflammation remain unclear. They have been predominantly reported in animals^5–7^ and rarely in humans.^8^ AlOOH in the nanoparticulate form can undergo cell-mediated migration from these granulomas to regional lymph nodes and translocate systemically, including to the central nervous system.^4,5^ Al has been linked to several adverse effects, including persistent granulomas,^5,9^ autoimmune/autoinflammatory conditions in humans and sheep,^4,10^ IS-associated sarcomas in animals,^11^ or neurological disorders,^4^ among others.

Bluetongue, a devastating midge-borne infection affecting sheep caused by bluetongue virus (BTV), is constantly reemerging in Europe since the beginning of the 21st century.^12^ Al-containing vaccines against BTV are included in sanitary programs, successfully controlling the infection^13^ but adding more Al-vaccines to the routine sanitary sheep schedules.^14^ Intensive vaccination, including AlOOH-based BTV vaccines, has been associated with neurological, reproductive, dermatological, and wasting disorders.^10,15^

Alternative biodegradable adjuvants to Al could be of great interest if similar immunization efficacy and increased safety profiles could be demonstrated.^1,16–18^ Ideally, alternatives should be cost-effective, noncumulative biomaterials with minimal environmental impact.^16^ A promising candidate is apatite nanoparticles (ApNPs), which resemble the calcium phosphate of mammalian bones and teeth, making them nontoxic, biocompatible, and biodegradable.^19^ ApNPs in the form of calcium-deficient hydroxyapatite were successfully used in the 1960s as an adjuvant for several vaccines, but manufacturing challenges led to their exclusion by the late 1980s.^18^ Advances in nanotechnology have renewed interest in their potential as next-generation immunostimulants.^20^ Modern ApNPs, engineered for more precisely controlled size and morphology, elicit robust and balanced Th1/Th2 immune responses while maintaining high safety standards and less IgE levels than AlOOH. Some have shown no toxicity or local inflammation in human trials.^20,21^ Although never previously tested, biomimetic ApNPs have recently been proposed as customizable and potentially superior vaccine adjuvants.^22^ Biomimetic approaches integrate natural bone ions (e.g., citrate, carbonate, sodium) into ApNPs, enhancing their bioactivity, biocompatibility, and protein adsorption capacity.^23,24^ Despite their extensive use in bone engineering, drug/biomolecule delivery, and imaging,^24^ their efficacy as a vaccine adjuvant remains unproven.^22^ Another promising biodegradable adjuvant is microcrystalline tyrosine (MCT), which has been used in human allergen-specific immunotherapy since the 1970s.^17,25^ Extensive toxicological studies and clinical evidence have confirmed its safety, with no reported adverse reactions in humans or animal models.^26^ MCT induces Th2 immunity but with a stronger Th1 response compared to AlOOH, less IgE, and adverse events in anaphylactic models.^27^ Additionally, MCT has demonstrated efficacy as an adjuvant in prophylactic vaccines and as a versatile platform in synergistic adjuvant systems.^25^

ApNPs and MCT have been previously proposed as alternatives to traditional Al-based adjuvants,^16,17^ but no research has simultaneously evaluated ApNPs, MCT, and AlOOH adjuvants in a direct comparison. This study provides an assessment of the immunogenicity and local safety of BTV vaccine prototypes formulated with AlOOH, biomimetic ApNPs, or MCT in a sheep model. The results show that ApNPs and MCT can induce serological responses comparable to or greater than those elicited by AlOOH, while they cause only mild and transient local inflammation. These findings suggest that ApNPs and MCT may represent promising candidates for inclusion in next-generation vaccines with improved safety profiles.

## 2 Results

### 2.1 Physicochemical Properties of Adjuvants and Virus

The AlOOH adjuvant (Adjuval) consisted of small, thin, mildly laminated, aggregated curved particles, characteristic of amorphous structures (Figure 1A-B). Due to the intensity of aggregation, only the size of aggregates and the thickness of particles could be measured by electron microscopy (TEM and HAADF-STEM) (Figure 1C). Laser diffraction analysis (LDA) revealed a trimodal aggregate distribution for the AlOOH-only adjuvant in PBS, whereas AlOOH/BTV exhibited a more homogeneous, monomodal population resembling the second peak (mode 2, M2) of the AlOOH-only distribution (Figure 1D). The AlOOH adjuvant used in this work (Adjuval) displayed broader and low-intensity XRD peaks similar to those of Alhydrogel (a standard AlOOH adjuvant), both exhibiting a diffractogram compatible with poorly crystallized boehmite (pseudoboehmite) (Figure S1A). The morphology of particles from both Al adjuvants appeared as nanoplate-like structures, with Adjuval particles being slightly thinner and more laminated (Figure S1B–C). ApNPs were characterized as short, rod-shaped nanoparticles (Figure 1E-F) with a high aspect ratio (AR) (Figure 1G), presenting a homogeneous monomodal distribution of micron-sized aggregates (Figure 1H). Upon vaccine formulation, ApNPs aggregates in solution increased in size, as indicated by a rightward shift and higher values across all LDA parameters (Figure 1H; Table S1). This increase was statistically significant (*p* < 0.001) in distribution span (heterogeneity) and coarse aggregate size, with elevated Dv90 values (Table S1). ApNPs displayed characteristic diffraction reflections of the hydroxyapatite single phase (ICDD 9–432), with broad diffraction peaks indicative of limited crystallinity (Figure S1D). MCT consisted of heterogeneous, elongated lath-shaped, micron-sized particles that were birefringent under polarized light and composed of stacked nanometric laminae (Figure 1I-J). These structures exhibited a monomodal yet heterogeneous particle size distribution, within the measurement range of optical microscopy and SEM (Figure 1K-L). Notably, no significant changes were observed after the formulation of MCT with the virus antigen, as measured in solution by LDA (Figure 1L). The XRD diffractogram of MCT corresponded to a highly crystalline form of the amino acid l-tyrosine (Figure S1E). All three adjuvants and the virus solution containing the inactivated BTV serotype 4 (BTV-4) exhibited a negative ζ potential in PBS. Among them, ApNPs aggregates showed a significantly lower ζ potential (−18.0 ± 0.6 mV) compared to AlOOH (−11.4 ± 0.5 mV) and MTC (−10.3 ± 0.6 mV) (*p* = 0.01). In addition, ApNPs was the only adjuvant with a significantly (*p* = 0.01) lower ζ potential than the viral protein solution (−11.2 ± 0.6 mV) (Table S1).

**Figure 1:**
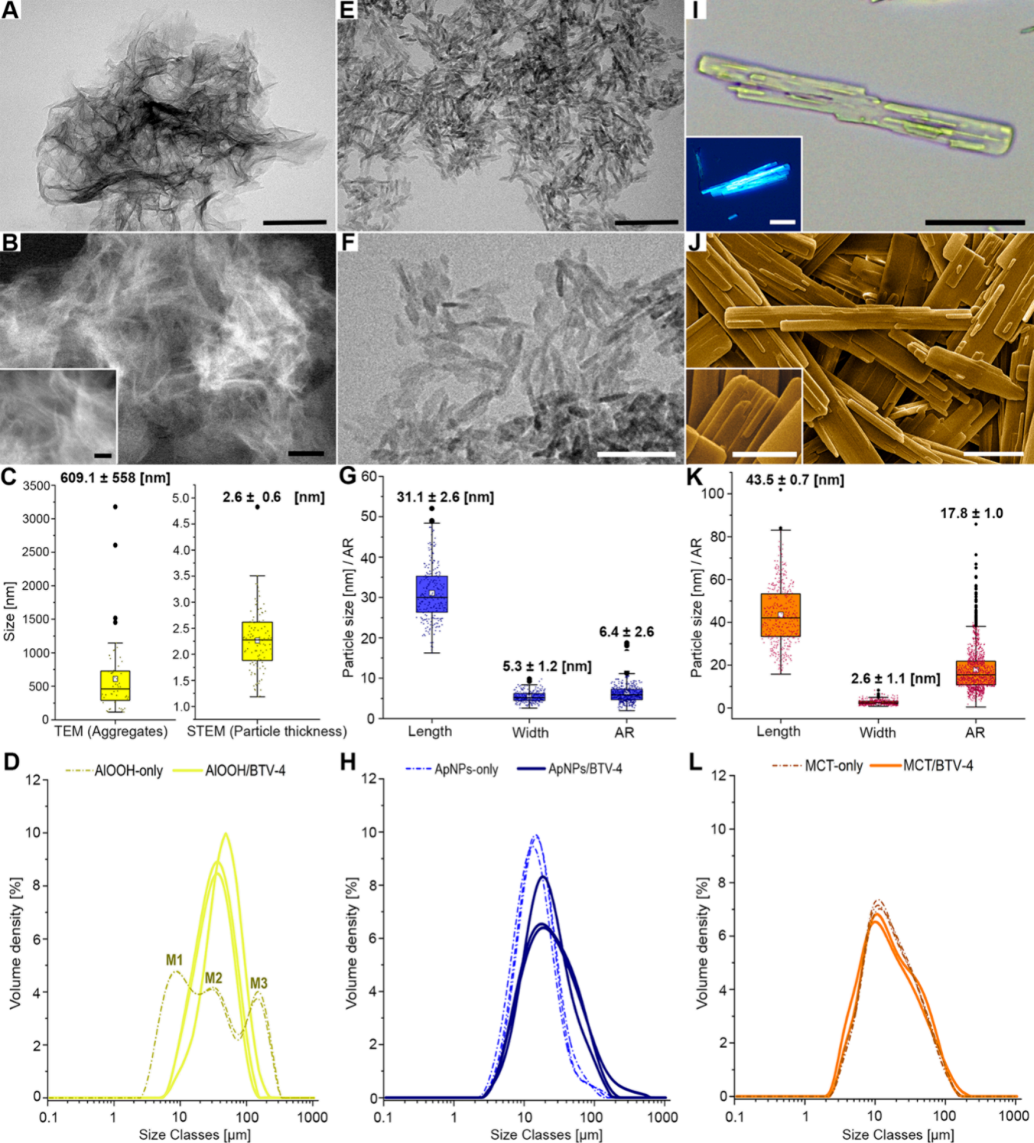
Physicochemical characteristics of **(A-D)** AlOOH (Adjuval); **(E-H)** Biomimetic apatite (ApNPs), and **(I-L)** Microcrystalline tyrosine (MCT). **A-B**. AlOOH adjuvant in a sample of 0.01 mg mL^–1^. **A.** TEM image of the AlOOH aggregates. Bar: 100 nm. **B.** STEM image of AlOOH aggregates. Bar: 50 nm. *Inset*: detail of thinner pseudoboehmite nanoparticles. Bar: 20 nm. **C.** Size distribution of aggregates by TEM and thickness of particles by STEM in a sample of AlOOH. **D.** Laser diffraction analysis (LDA). Size distribution of AlOOH aggregates in PBS (AlOOH-only; 6 mg mL^–1^) and in vaccine (AlOOH/BTV-4); M1, mode 1; M2, mode 2; M3 mode 3. **E-F.** TEM image of ApNPs aggregates in a sample of 0.01 mg mL^–1^. **E.** ApNPs nanoplates. Bar: 100 nm. **F.** Detail of the rod-like nanoplates of ApNPs. Bar: 50 nm. **G.** Length, width, and aspect ratio (AR) distribution of ApNPs. **H.** LDA. Size distribution of ApNPs aggregates in PBS (ApNPs-only; 6.25 mg mL^–1^) and in vaccine (ApNPs/BTV-4). **I.** Optical microscopy of MCT crystals in a PBS solution. Unstained section. Bar: 20 μm. *Inset*: birefringence of MCT crystals under polarized light. Bar: 20 μm. **J.** SEM image of MCT crystals. Bar: 5 μm. *Inset*: detail of the stacked laminae. *Inset bar*: 2 μm. **K.** Length, width, and AR distribution of MCT particles. **L.** LDA. Size distribution of MCT aggregates in PBS (MCT-only; 20 mg mL^–1^) and within MCT vaccine (MCT/BTV-4).

### 2.2 Group Specific AntiVP7/BTV Antibodies

Antibody levels across groups and the effects of prime and booster, expressed as absorbance values (O.D. 450 nm), are summarized in Figure 2, with the complete data set provided in Table S2 and Table S3. In all groups, antibody levels were significantly higher (*p* < 0.001) across most study time points than those measured at 0 days postprime inoculation (DPI), that included data from all animals and served as the experimental baseline (EB). Exceptions were observed at 28, 35, and 42 DPI for the AlOOH group and at 14 and 21 DPI for the MCT group, where no significant increase was detected. Most groups exhibited significant (*p* < 0.001) increases in antibody levels at any week compared to the PBS group. After prime immunization (7 DPI), antibody levels were significantly elevated (*p* < 0.001) in all vaccinated groups (Figure 2A-D), with a 17-fold increase for BTV-4, a 16-fold increase for AlOOH, 13-fold increase for ApNPs, and 7-fold increase for MCT compared to EB. At 7 DPI, antibody levels in the AlOOH, BTV-4, and ApNPs groups were significantly higher (*p* < 0.001) than those of MCT (Figure 2E). All groups showed a progressive decrease in antibody levels from 7 to 21 DPI, except for the AlOOH group, which continued to decrease until 35 DPI. The lowest antibody level value was observed in the MCT group at 21 DPI (Figure 2D). After booster immunization (21 DPI), antibody levels showed two different patterns of increase. The first pattern, represented by BTV-4 and AlOOH groups, was characterized by a delayed response, with a gradual serological increase persisting until the end of the study. Remarkably, in the AlOOH group after booster, there were no significant serological responses at any DPI, despite a progressive increase in antibody levels. The second group, represented by ApNPs and MCT, showed an immediate response to the booster, followed by a sustained antibody plateau until the end of the experiment. The ApNPs group showed a significant (*p* = 0.031) response to the booster at 28 DPI, whereas the MCT group demonstrated a significant response to the booster at 28 (*p* = 0.009) and 35 (*p* = 0.043) DPI. Antibody levels continued to rise over time, with a significant booster effect, persisting until the end of the experiment, observed from 70 DPI in the MCT group and from 77 DPI in the BTV-4 and ApNPs groups. At the end of the study (133 DPI), the ApNPs group exhibited significantly (*p* < 0.001) higher antibody levels compared to other vaccinated groups, among which no significant differences were observed (Figure 2E).

**Figure 2:**
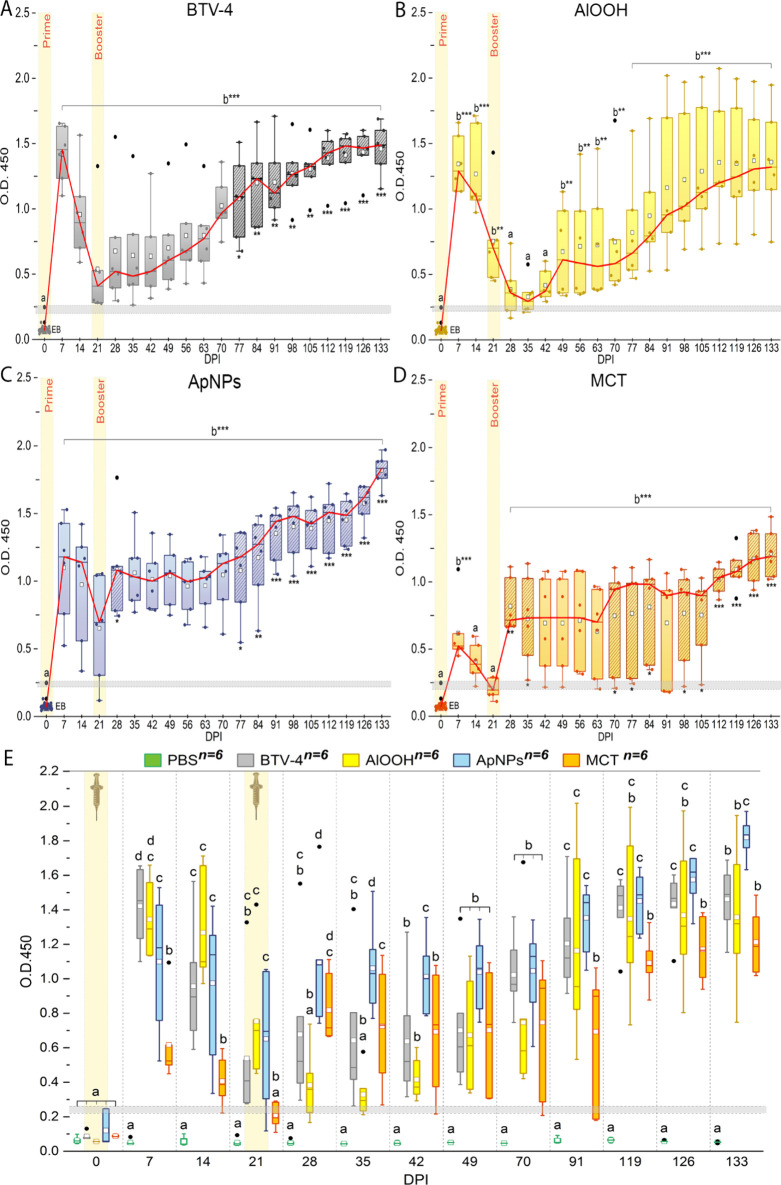
Evolution of antibody levels expressed as absorbance (O.D. 450) in (**A**) the BTV-4, (**B**) ApNPs, (**C**) AlOOH, and (**D**) MCT groups and (**E**) comparison between groups at each day postprime inoculation (DPI). **A-E**. Boxplots show interquartile ranges of absorbance values for animals (*n* = 6) at each DPI with medians (red line), means (white squares), and whiskers for min and max values, excluding outliers (black dots). The gray band represents cutoff range (15% of absorbance of the control positives on each plate); values within or below were considered negative. **A-D**. Data for all groups (*n* = 30) at 0 DPI is denoted as experimental baseline (EB). Significant differences from EB are indicated over the boxplots, with letters. Significant differences with 21 DPI (booster date) are indicated as patterned boxplots, and *p* values are expressed below these boxplots. *p < 0.05, **p < 0.01, ***p < 0.001. **E**. Groups with different letters indicate significant differences (***p < 0.001). Data from 56, 63, 77, 84, 98, 105, and 112 DPI were excluded due to similarity with earlier time points.

### 2.3 Neutralizing Antibodies against BTV-4 and Cross-Neutralization with BTV-1 and BTV-8

Figure 3 indicates neutralizing antibody titers and the comparison between groups and DPI. The complete data set is presented in Table S4 and Table S5, respectively. Median values for antibody titers did not vary between DPI in the PBS group. All groups exhibited significantly higher antibody titers than the PBS group at 35, 70, 119, and 133 DPI, except for the AlOOH group at 35 DPI where titers were significantly lower (*p* = 0.006) than those in other groups. Titers in the ApNPs and MCT groups showed significant (*p* = 0.003) increases at 35 DPI compared with the AlOOH group, an increase also detected for the BTV-4 group (*p* = 0.032). Titers in the AlOOH group increased significantly (*p* = 0.016) thereafter. The BTV-4 group showed a significant increase in titers (*p* = 0.028) at 119 DPI, followed by a significant decrease (*p* = 0.027) at 133 DPI. Titers were maintained throughout the study for the ApNPs group but significantly decreased (*p* = 0.005) during the study for the MCT group. BTV-4, ApNPs, or MCT groups did not show significant differences in neutralizing titers with the AlOOH groups at 70, 119, and 133 DPI. However, BTV-4 and ApNPs groups showed significantly (*p* = 0.020 and p = 0.003, respectively) higher titers at 133 DPI, compared with MCT. None of the groups induced cross-neutralizing antibodies against BTV-1 and BTV-8 serotypes (Figure S2).

**Figure 3:**
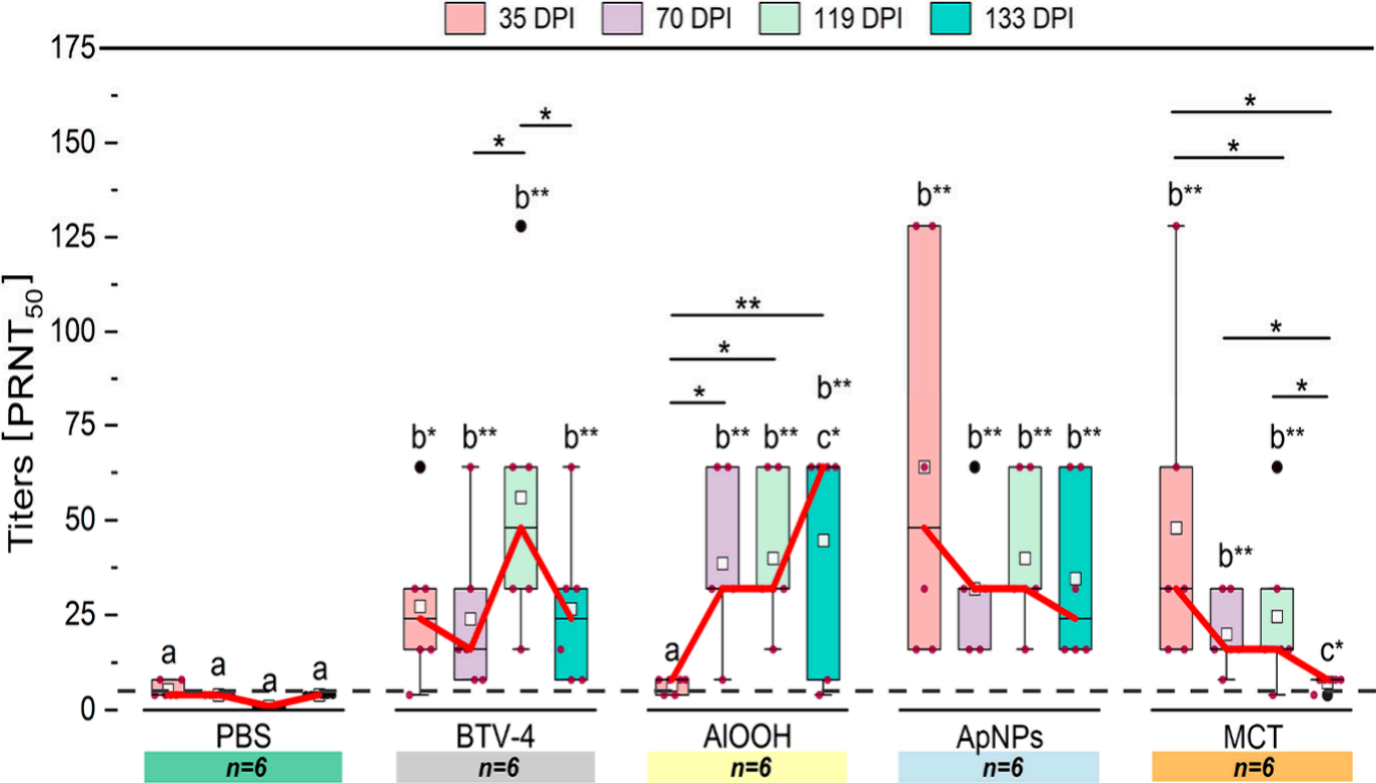
BTV-4 neutralizing antibody titers per group and date. Boxplots show interquartile ranges of titers (PRNT_50_) at each DPI, with mean (white square), median (horizontal lines), and whiskers representing minimum and maximum values, excluding outliers (black dots). Different letters denote significant differences between groups per DPI; significant differences within groups per DPI are indicated with a line (above). Red line: median. Dash line: cutoff (1:5). *p < 0.05*;* **p < 0.01; ***p < 0.001.

### 2.4 Complete Blood Cell Counts and Clinical Chemistry

Mean values for most hematological (Table S6 and Table S7), biochemical (Table S8), and exploratory clinical analyses (Table S9) remained within normal ranges or followed trends like the PBS group. Median monocyte counts were significantly higher (*p* < 0.001) in the BTV-4, AlOOH, and MCT groups than in PBS (Figure S3A). Additionally, monocyte counts in the BTV-4 and AlOOH groups were significantly elevated (*p* < 0.001) compared to the ApNPs group, which remained similar to the control. Temporal analysis showed no significant intra- or intergroup differences in monocyte kinetics. AlOOH showed a biphasic monocytosis, with peaks at 21 and 84 DPI, followed by declines at 63 and 112 DPI (Figure S3B). In contrast, the BTV-4 group exhibited a delayed monophasic monocytosis at 112 DPI, followed by a rapid decrease at 133 DPI. Only the MCT group exhibited a significant time-dependent increase in Tbil levels that were significantly higher (*p* = 0.042) at 133 DPI compared to both baseline and levels measured 1 week after primovaccination (Figure S3C).

### 2.5 In Vivo Local Assessment of Injection Site (IS) Reactions of Vaccines

Palpation results are depicted in Figure 4 and Figure 5 for the prime inoculation and booster doses, respectively. The statistical analysis is provided in Table S10 and Table S11. The chi-square (χ^2^) test was used to compare observed data with the expected frequency, assuming conditional independence between groups and IS reactions. AlOOH-induced reactions manifested as nonvisible, well-defined subcutaneous nodules, either round or ovoid. Reactions to ApNPs and MCT were characterized by nonvisible, poorly demarcated subcutaneous indurations. No IS reactions were palpated in PBS and BTV-4 groups (nonadjuvanted formulations) (Figures 4A and 5A). After prime inoculation (Figure 4), the AlOOH group exhibited the highest and most severe proportion of IS reactions, which were significantly (*p* < 0.001) associated with vaccination (Figures 4B and 4E), differences between groups persisting up to 117 DPI (Table S10). The AlOOH group exhibited a significantly higher-than-expected frequency of grade 3 reactions within the first 15 DPI (*p* < 0.001; Figure 4B). Grade 2 reactions were also more frequent than expected at 22–25 DPI (*p* < 0.001; *p* < 0.05) and 35–38 DPI (*p* < 0.05), while grade 1 reactions occurred more frequently than expected from 31 to 117 DPI (*p* < 0.01). Notably, 33.3% (2/6) of grade 1 reactions in the AlOOH group persisted until the conclusion of the study (Figure 4B). Reactions observed with ApNPs (Figures 4C and 4E) and MCT (Figures 4D and 4E) were significantly less frequent than expected, resolving around 18 and 13 DPI, respectively. All grades, including grade 3 reactions, were significantly (*p* < 0.001) less frequent than expected in these groups (Figures 4C–D and 4E). After booster inoculation (Figure 5), AlOOH group reactions (mostly grade 1) remained significantly more frequent than expected (*p* < 0.001) and were still palpable at the end of the experiment (Figures 5B and 5E). Booster reactions with ApNPs (Figures 5C and 5E) and MCT (Figures 5D and 5E) varied in grade but generally exhibited a nonsignificant trend toward higher grades compared to AlOOH and those observed during primovaccination. These reactions resolved by 49 DPI (28 days postbooster; Figure 4D). In the ApNPs group, following the booster dose, one animal developed a visible erythematous indurated area, classified as grade 3. This area was warm, decreased in size by nearly half within the first 10 days, and healed completely by 41 DPI (Figure S4).

**Figure 4:**
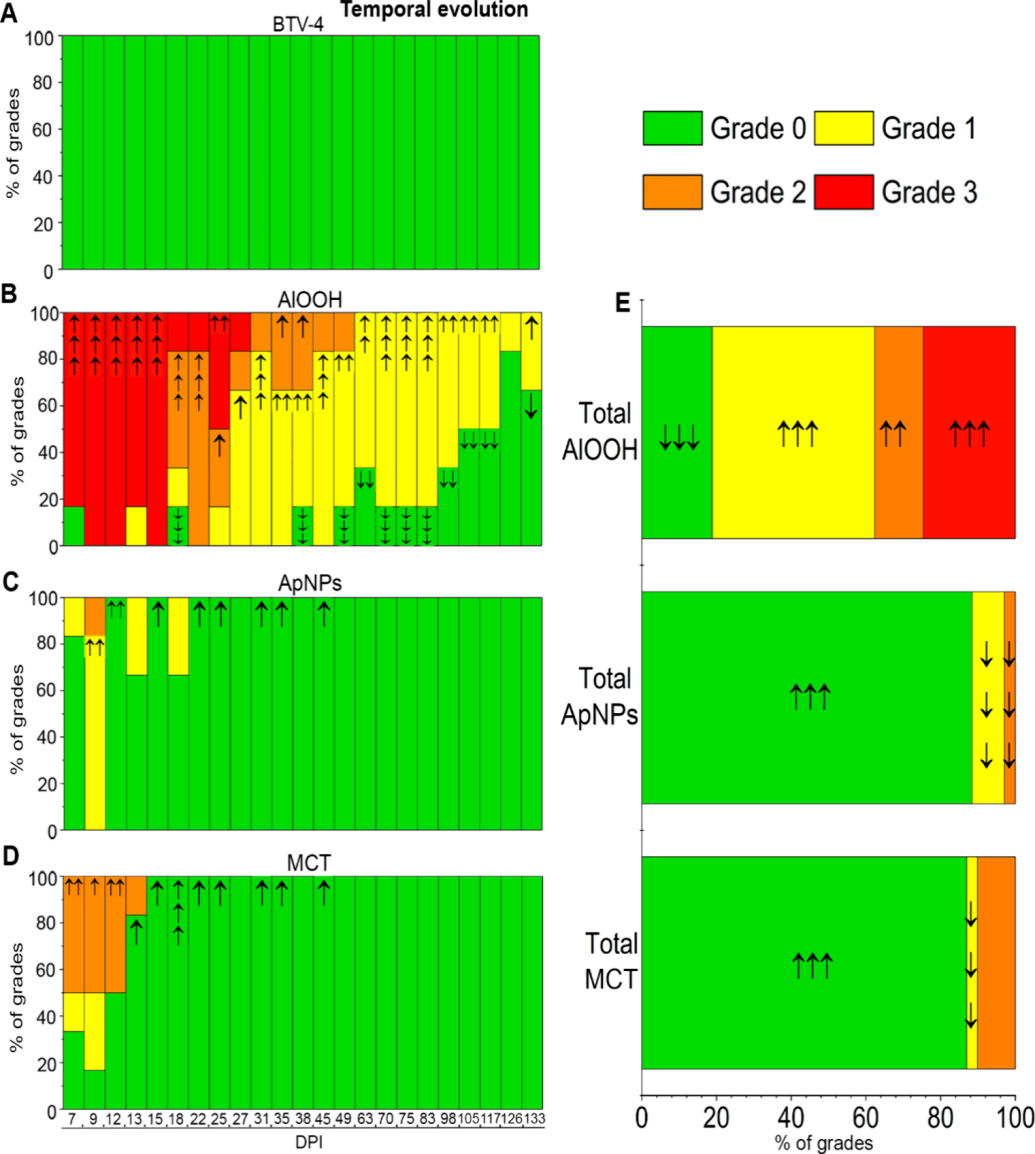
Prime inoculation. Relative frequencies of injection site (IS) reactions per group throughout the experiment (**A**-**D**) and total frequencies (**E**). Grades of severity are represented by colors from grade 0 (not found) to grade 3 (large). The presence of arrows indicates statistical significance according to the likelihood ratio test. Arrows indicate the direction and magnitude of significance based on standardized adjusted residuals: one arrow (*p < 0.05), two arrows (**p < 0.01), three arrows (***p < 0.001). *DPI*, day postprime inoculation.

**Figure 5:**
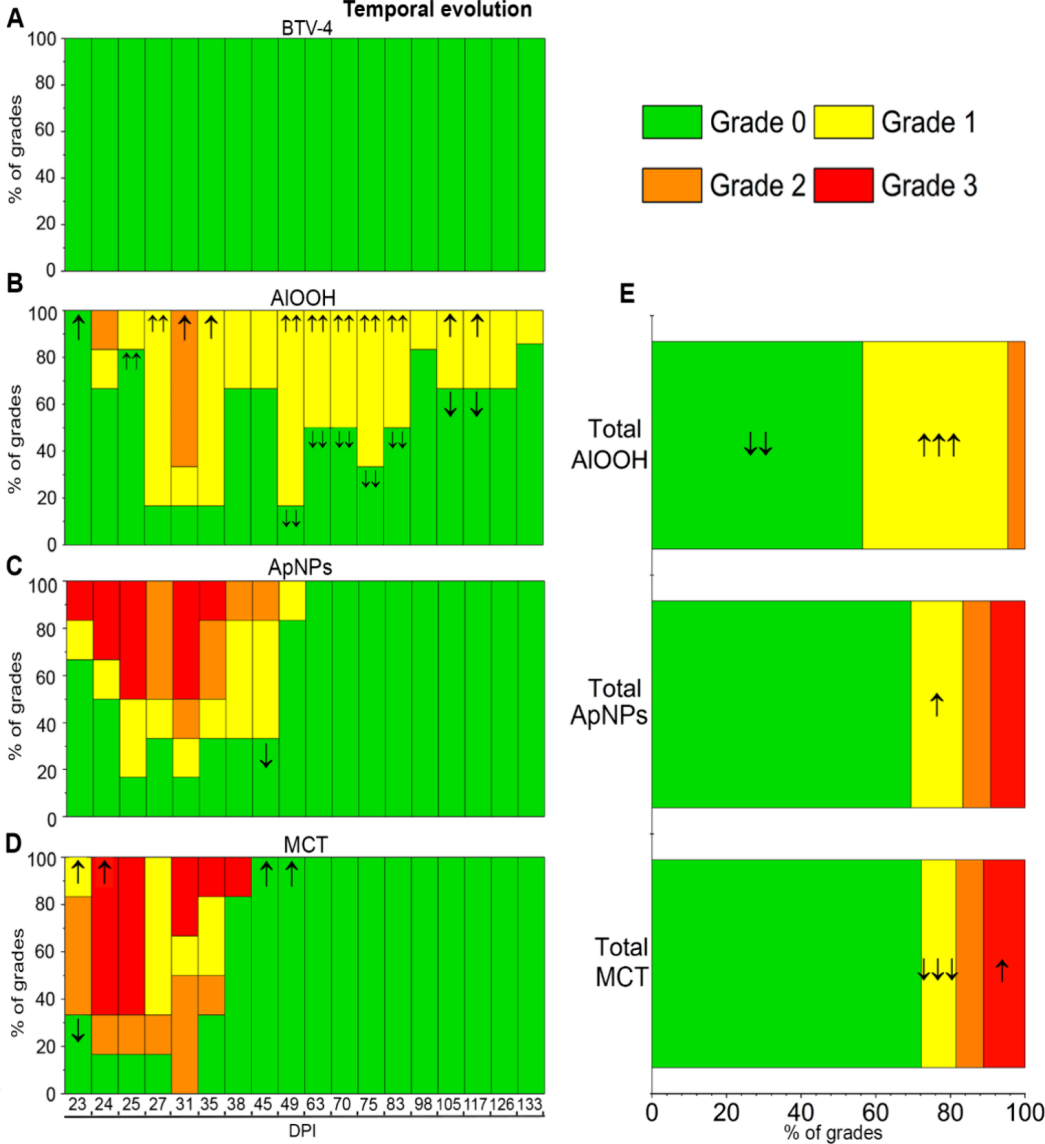
Booster inoculation. Relative frequencies of injection site (IS) reactions per group throughout the experiment (**A**-**D**) and total frequencies (**E**). Grades of severity are represented by colors, from grade 0 (not found) to grade 3 (large). The presence of arrows indicates statistical significance according to the likelihood ratio test. Arrows indicate the direction and magnitude of significance based on standardized adjusted residuals: one arrow (*p < 0.05), two arrows (**p < 0.01), three arrows (***p < 0.001). *DPI*, day postprime inoculation.

### 2.6 Post-Mortem Findings

Only the AlOOH group exhibited gross and histopathological reactions at the IS reactions post-mortem; their clinicopathological findings are summarized in Table 1 and Figure 6. Only 33.3% of the reactions were palpated in vivo but up to 83.3% exhibited subcutaneous granulomas at necropsy. In both primovaccination and booster, 5 out of 6 IS reactions were found, totaling 10 AlOOH granulomas out of 12 inoculations (83.3%).

**Table 1:** AlOOH Group^a^

	***n***	Palpated nodules	Granulomas at necropsy	Voluminous granulomas	Flattened granulomas	Gross necrosis in granuloma	Microscopic necrosis
Prime ISs^a^	6	33.3% (2/6)	83.3% (5/6)	80.0% (4/5)	20.0% (1/5)	80.0% (4/5)	80.0% (4/5)
Booster ISs^a^	6	33.3% (2/6)	83.3% (5/6)	20.0% (1/5)	80.0% (4/5)	0.0% (0/5)	20.0% (1/5)
Total	12	33.3% (4/12)	83.3% (10/12)	50.0% (5/10)	50.0% (5/10)	40.0% (4/10)	50.0% (5/10)

a Clinicopathological findings of post-vaccination granulomas at 133 days post-inoculation.

b ISs, injection sites.

**Figure 6:**
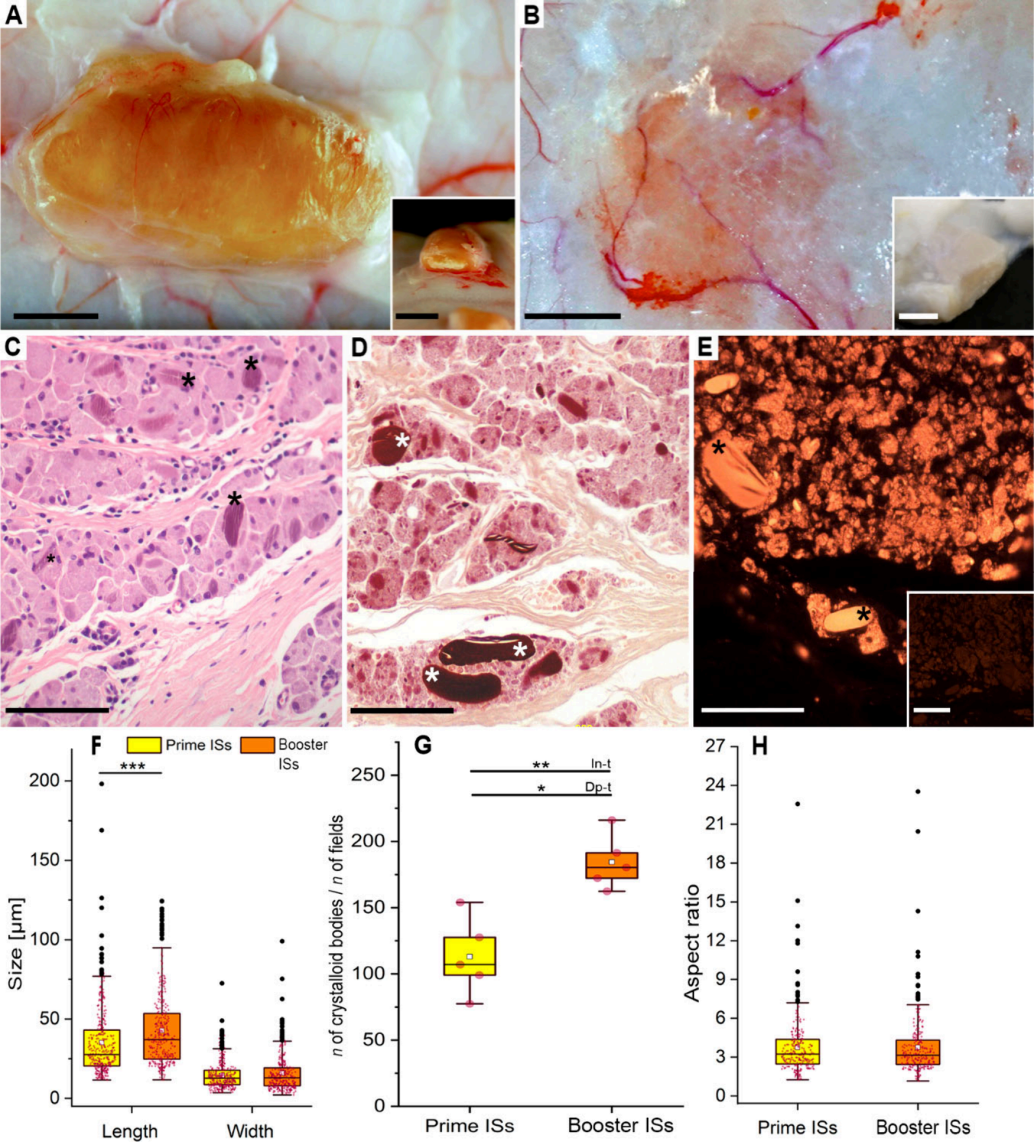
AlOOH Group, pathological findings in postvaccination granulomas. **A.** Large, voluminous AlOOH granuloma mostly associated with prime inoculation. Inset: The same granuloma showed a necrotic center after sectioning. Both bars: 1 cm. **B.** Flat AlOOH granuloma were mostly associated with booster inoculation. Inset: The same granuloma does not exhibit a necrotic center (fixed specimen). Bar 1 cm; Inset bar: 0.5 cm. **C.** AlOOH-induced granuloma, showing granular macrophages and crystalloid bodies (black asterisks). HE, Bar: 100 μm. **D.** Granular macrophages and crystalloid bodies (white asterisks) are stained deep purple. Pale-yellow fibrous tissue bands within the granuloma serve as an internal negative control. Modified aluminum-hematoxylin; Bar: 100 μm. **E.** Aluminum within macrophages and crystalloid bodies (black asterisks) is observed as gold-orange fluorescence. Inset: negative control. Lumogallion stain. Bar 100 μm; Inset bar: 50 μm. **F–H.** Width, length, counts, and aspect ratio of crystalloid bodies of AlOOH granulomas. Boxplots show interquartile ranges, medians (horizontal lines), means (white squares), and whiskers indicate minimum/maximum values, excluding outliers (black dots). **F.** Length and width distribution of crystalloid bodies in prime and booster inoculation granulomas. ***p < 0.001. **G.** Distribution of indices is based on the number of crystalloid bodies per field in prime and booster inoculation granulomas. In-t, Independent-samples Student’s *t* test; Dp-t, paired *t* test; *p < 0.05; **p < 0.01. **H.** Aspect ratio of crystalloid bodies in prime and booster inoculation granulomas.

Most granulomas from prime inoculation were firm, well-defined, voluminous ovoid masses measuring 1–2 cm, characterized by slight ochre discoloration and caseous necrotic centers (Figure 6A). Most granulomas from booster inoculation were observed as flattened, less voluminous nodules without necrotic centers (Figure 6B). Microscopically, 50% of granulomas exhibited central necrosis, with no bacteria (including acid-fast bacteria) or fungal agents detected by histopathologic stains. Granulomas displayed encapsulated infiltrates of large, granular, uni- to multinucleated macrophages, accompanied by intra- and extracytoplasmic AlOOH crystalloid bodies (Figure 6C). These cytoplasmic granules and crystalloid bodies were Al-positive, exhibiting a characteristic deep purple staining with modified aluminum-hematoxylin stain (MAH) (Figure 6D) and a golden-orange fluorescence with lumogallion while showing no detectable autofluorescence in unstained sections (Figure 6E). Crystalloid bodies in booster inoculation granulomas were significantly longer (36.76 ± 28.94 μm) than those in prime inoculation granulomas (26.66 ± 20.10 μm) (*p* < 0.001; Figure 6F). The number of crystalloid bodies was significantly increased on the booster inoculation granulomas (Index = 184.50 ± 20.60) compared to the prime inoculation granulomas (Index = 113.03 ± 29.11) (independent-samples Student’s *t* test: *p* = 0.002 and paired *t* test: *p* = 0.017; Figure 6G). No significant differences were noted in the width or AR (Figures 6F and 6H). No gross or microscopic findings were observed at ISs of ApNPs and MCT groups. No other histopathological changes were observed in any animal from the five groups.

A comprehensive study was conducted on histological changes associated with lymphoid hyperplasia in prescapular and axillary lymph nodes, including cortex-paracortex thickening (CPT), secondary follicles (SF), medullar plasmacytosis (MP), medullar histiocytosis (MH), and follicular hyalinosis (FH) (see the Experimental Section). Results are summarized in Figure 7 and Table S12. Regarding the global lymphoid hyperplasia score, the AlOOH group (8.88 ± 3.08) had a significantly (*p* = 0.001) higher total lymphoid hyperplasia score than PBS (7.29 ± 1.99), BTV-4 (7.46 ± 2.23), and ApNPs (6.13 ± 1.70), while MCT (8.55 ± 2.34) showed no significant difference when compared with AlOOH (Figure 7A). Scores for each microscopic change per group indicated similar radar chart patterns (Figure 7B), with the largest areas corresponding to AlOOH and MCT groups, followed by the BTV-4 group; ApNPs exhibiting the smallest area. CPT and SF were significantly more severe in the AlOOH and MCT groups than in ApNPs (Table S12). Macrophage aggregates were observed in all sheep groups with a significantly higher frequency of detection (*p* < 0.001) in lymph nodes from the AlOOH group compared to the ApNPs and MCT groups (Figure 7C and Table S13). Macrophage aggregates in the AlOOH group were significantly more frequent (*p* < 0.001) in lymph nodes from the prime inoculation than those from the booster side. In the ApNPs group, macrophage frequency was significantly lower (*p* < 0.001) in lymph nodes from the prime inoculation site (0/12; 0%), consistent with mild local lesions (Table S13). Only in the lymph nodes of the AlOOH group were these macrophage aggregates shown Al and crystalloid bodies (Figure 7D); most axillary lymph nodes were positive for MAH (7/12; 58.33%), whereas the majority of prescapular lymph nodes was negative (1/12; 8.33%). No other macrophage aggregate in any other experimental group was positive for Al specific stains. Remarkably, the frequency of Al-laden macrophage aggregates in the right axillary lymph node (prime-sided) was only significantly (*p* = 0.0497; Index = 1.50 ± 2.31) higher than that in the left axillary lymph node (booster-sided) (Index = 0.01 ± 0.02) when analyzed independently (Figure 7E).

**Figure 7:**
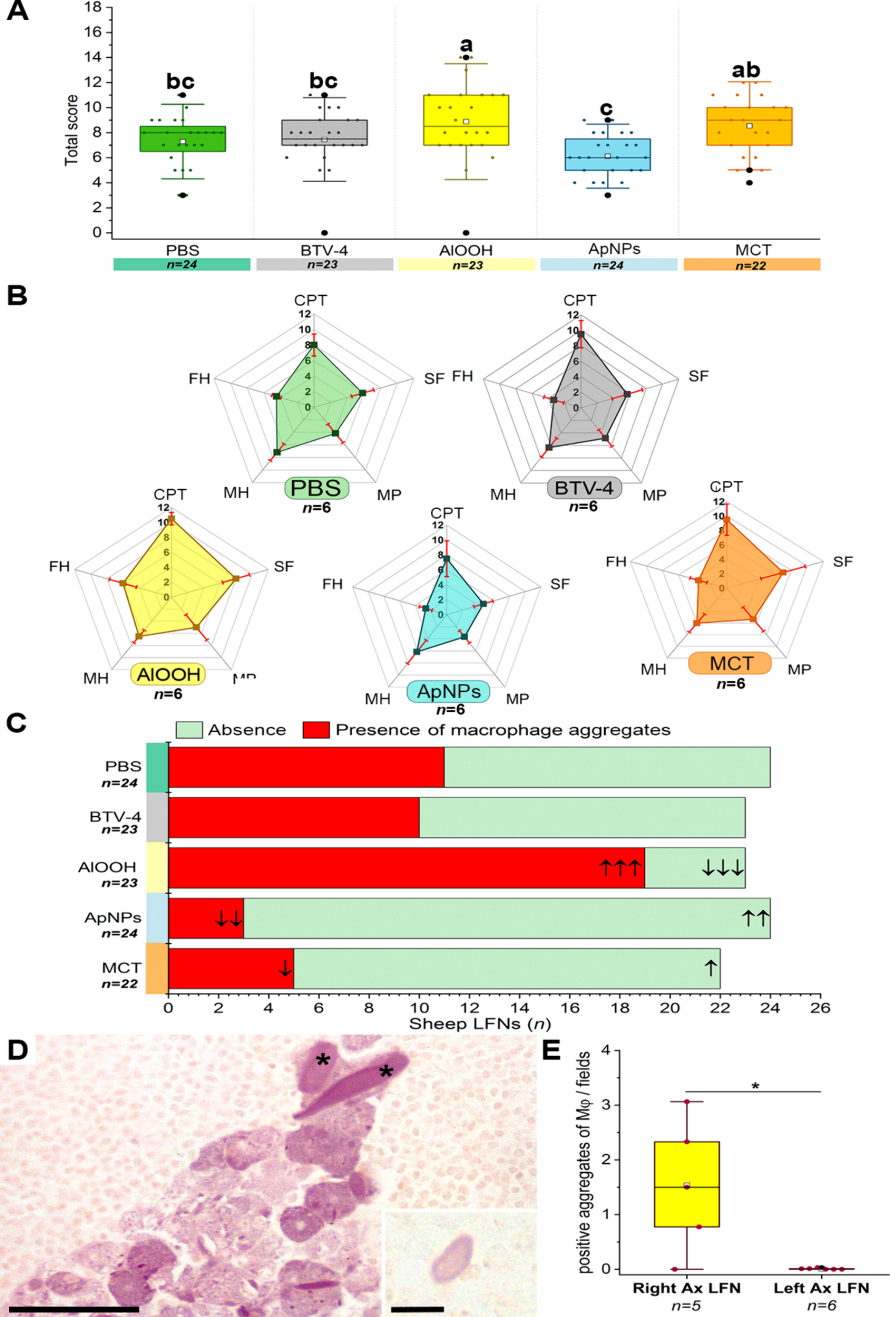
**A-B.** Results of lymphoid hyperplasia changes in regional lymph nodes by group. **A.** Total lymphoid hyperplasia scores. Different letters indicate significant differences (*p* = 0.001). **B.** Scores for each histological change (Mean ± SD; *n* = 6). CPT, cortex-paracortex thickening; SF, secondary follicles; MP, medullary plasmacytosis; MH, medullary histiocytosis; and FH, follicular hyalinosis. **C-E.** Lymph nodes studies. **C.** Presence of aggregates of macrophages per group by HE. Arrows indicate statistical significance according to the likelihood ratio test (prime-sided) or chi-square test (boost-sided and total). Arrows also indicate the direction and magnitude of significance based on standardized adjusted residuals: one arrow (*p < 0.05), two arrows (**p < 0.01), three arrows (***p < 0.001). **D.** AlOOH group. Macrophage aggregates exhibit intracellular, deep purple Al deposits and crystalloid bodies (asterisks). Modified Al-hematoxylin (MAH), bar: 50 μm. Inset: extracellular crystalloid bodies. MAH, Inset bar: 10 μm. **E.** Positive Al-laden macrophage in axillary lymph nodes following the prime inoculation (right) and booster (left), Kruskal–Wallis (KW). Ax LFN, axillary lymph node; Mφ, macrophages; *p < 0.05.

## 3 Discussion

This study provides a comprehensive assessment of three adjuvants in BTV vaccine prototypes for sheep, demonstrating that both ApNPs and MCT elicit potentially protective antibody responses without inducing persistent local reactions, thereby enhancing safety compared to AlOOH. To our knowledge, no previous study has directly compared these adjuvants in parallel using the same antigen and species. Our findings support the potential of ApNPs and MCT are safer adjuvant alternatives for veterinary vaccines and promising results for human vaccines.

AlOOH has been used as a vaccine adjuvant for nearly a century, and it remains common in commercial formulations. However, AlOOH has been associated with adverse effects in multiple species, but scientific data on these effects remain limited.^1,4,5^ Safer alternatives capable of eliciting comparable protection with fewer side effects are therefore needed.^16^ To evaluate such alternatives, we employed a model based on BTV, the etiology of a severe ovine disease that has affected European livestock since the early 2000s.^12^ In Europe, the only licensed vaccine for BTV relies on serotype-specific inactivated antigen adjuvanted with AlOOH and Quil A,^12^ with AlOOH playing a key role in the induction of persistent local granulomas at ISs.^5^ Despite increasing focus on next-generation BTV vaccines, research into biodegradable, less reactogenic adjuvants remains scarce. ApNPs and MCT elicited antibody responses against BTV without causing persistent local reactions, thereby enhancing vaccine safety.

Rational design of adjuvant-antigen systems is essential in vaccine design, as physicochemical properties govern biodistribution, cytotoxicity, and immune outcomes.^28^ Adjuval (CZ Vaccines) exhibited physicochemical profiles comparable to Alhydrogel,^28–30^ differing mainly in thinner laminar particles, likely contributing to its broader 020 XRD peak (Figure S1B–C).^31^ Upon protein addition, Adjuval shifted from a multimodal to monomodal size distribution, suggesting homogenization rather than aggregate growth (Figure 1D). Alhydrogel aggregates responded similarly, though resulting aggregates appeared larger.^28,29^ PBS probed a suitable vehicle for protein-AlOOH interaction, despite ζ potential data suggesting electrostatic repulsion. Its phosphate ions may modulate weak interactions,^32^ promoting disaggregation into phagocytosable sizes,^33^ possibly explaining the intracellular Al seen in macrophages at ISs and the efficacy of commercial BTV vaccines formulated in PBS. Phosphate-driven aggregation dynamics may confer immunologic benefits over NaCl,^34^ yet most AlOOH characterization reports still use NaCl.^28,29^ The ApNPs exhibited broad diffraction peaks indicative of single-phase, low-crystallinity nanoscale hydroxyapatite, consistent with the biomimetic ApNPs characterized elsewhere, resembling bone Ap.^23,24^ Their biomimetic compatibility was further supported by the match with the diffraction profile of ovine bone (Figure S1D). ApNPs microaggregates were larger and less homogeneous upon interaction with proteins included in the inoculum despite electrostatic repulsion between the negatively charged ApNPs and proteins, likely due to exposed positive calcium groups in the apatite interphase.^35^ To the best of our knowledge, this is the first demonstration of protein interaction with biomimetic ApNPs, supporting their potential as vaccine adjuvants. Bone-mimetic features may improve biocompatibility, solubility, protein adsorption, and sustained release over other hydroxyapatite-based ApNPs tested.^24,36^ The MCT adjuvant used in this work was compatible with l-tyrosine crystals (Figure S1E).^37^ Moreover, MCT exhibited stepped surfaces, reminiscent of the stacked nanolayers observed in self-assembled l-tyrosine microcrystals formed during precipitation in solution.^38^ No protein-MCT interaction was observed by laser diffraction (Figure 1L), consistent with previous studies.^37^ This lack of interaction might be due to the crystalline presentation of MCT, that shields hydroxyls groups, reducing hydrophilicity^38,39^ and water adsorption on MCT.^37^ However, MCT-allergoid interaction has been observed^40^ maybe through entrapment within crystal lattice,^41^ as precipitation takes place in the presence of antigen.^40^ In any case, the MCT size, not readily processed by cells, and its slow dissolution^37^ may facilitate depot formation and sustained antigen release, thereby demonstrating vaccine efficacy even in the absence of surface adsorption. Further investigation into MCT-protein interactions is needed.

The four vaccine prototypes used in this work (including BTV-4 virus only) successfully elicited a serological response, characterized by group-specific antibodies and sustained increases in antibody levels until the end of the experiment. Group-specific antibodies were detected with an ELISA validated for antibody kinetics either postvaccination or postinfection.^42,43^ Although the evolution of antibody levels showed a similar pattern following prime and booster, some differences between groups must be underlined. Seroconversion occurred as early as 7 DPI for all groups, paralleling results from other BTV vaccines^44^ and natural infections.^43^ BTV-4 and AlOOH groups showed the highest response after prime inoculation, although there were no differences between AlOOH and ApNPs groups. This early response decreased sharply for all groups, but it was less pronounced for ApNPs, which showed an early plateau between prime and booster vaccination. MCT showed the poorest reaction to a prime vaccination. After the booster, ApNPs and MCT groups responded immediately, whereas the response was delayed for BTV-4 and AlOOH groups. From 70 to 77 DPI onward, the serologic increase due to the booster was significant for BTV-4, ApNPs, and MCT groups, something not observed in the AlOOH group for the whole experiment. At the end of the experiment (133 DPI), the antibody response was similar in BTV-4, AlOOH, and MCT groups, but it was significantly increased in the ApNPs group. These results likely suggest the validity of all these vaccine prototypes to confer a similar immunogenicity to vaccinated animals, perhaps stronger using ApNPs. Remarkably, the BTV-specific antibody seroconversion in the nonadjuvanted group was similar to the AlOOH group, even with a significant increase in antibody levels after 77 DPI. Previous studies have also reported the induction of specific antibodies against certain BTV serotypes in sheep vaccinated without adjuvants,^45^ including neutralizing titers against BTV-4 in rabbits.^46^ These results indicate that inactivated BTV alone can elicit serological responses comparable to those induced with AlOOH and challenge the widespread misconception that inactivated BTV alone fails to generate an immune response. In this sense, previous trials studying adjuvants for BTV vaccines have not included a group with the inactivated virus alone, surely missing important information.^47,48^ These findings highlight the need for further studies evaluating whole inactivated BTV serotypes alone as potential vaccine candidates. Importantly, we analyzed the serological responses for 133 days only; the use of adjuvants in formulations may induce a longer immune response. Additional research is needed to clarify if all BTV serotypes can induce similar responses when only inactivated viruses are used and whether these responses are enough to confer long-term protection. However, the group injected with nonadjuvanted BTV-4 showed sharper antibody declines, unlike the more sustained, plateau-like kinetics seen with the other adjuvants. Boosting responses were also less immediate compared to ApNPs and MCT, suggesting that adjuvants modulate not only response magnitude but also its durability. Further studies with extended follow-up periods and larger sample sizes are needed to confirm these differences and their potential immunological relevance.

Neutralizing antibodies against BTV-4 were also produced by the four vaccinated groups, and titers were comparable between them, except for the reduced response in the AlOOH group at 35 DPI and the MCT group toward the end of the study. BTV-4 alone, ApNPs and MCT groups maintained an earlier and robust neutralizing response when compared with AlOOH. Nevertheless, BTV-4 alone elicited smaller increases in neutralizing titers over the control group than those induced by MCT and ApNPs at 35 DPI, with an abrupt rise followed by a declining trend toward the end of the study (though not falling below 35 DPI levels). At most time points, the highest individual titers were observed in animals receiving adjuvanted formulations. Only the AlOOH and MCT groups showed lower performance at the beginning or the end of the experiment, respectively. In previous studies, ApNPs were able to induce long-lasting antibody responses, in humans, mice, and chicken.^49–51^ Globally, these results indicate the capability of the four vaccine prototypes to induce neutralizing antibodies after vaccination, a response that was best maintained in the BTV-4 and ApNPs groups. However, a longer follow-up is necessary to better characterize the evolution and apparent decline noted in the BTV-4 group. The response lasted ∼3.5 months, aligning with the active season of *Culicoides imicola*, the main BTV-4 vector in Spain.^52,53^ Furthermore, the rapid induction of neutralizing responses observed in the BTV-4, ApNPs and MCT groups, compared to AlOOH, could offer a significant advantage for bluetongue control. Since vaccination is usually implemented after an outbreak is declared, eliciting a prompt protective response is crucial for effectively limiting viral spread.^54^ Although a challenge was not feasible in this experiment, a strong correlation between neutralizing antibodies and protection has been consistently demonstrated in sheep with similar neutralizing titers,^55–57^ and we can consider that all vaccinated groups were virtually protected against infection at most time points. For instance, neutralizing antibody titers as low as 1:16 (median) against BTV-4, measured by plaque reduction in groups of six sheep, conferred protection in 5 out of 6 animals.^56^ In cattle, titers as low as 1:10 at the time of high-dose challenge with BTV-4 similarly prevented viremia,^57^ and comparable titer ranges have also been reported as protective for other BTV serotypes, such as BTV-1 in sheep.^55^ No cross-neutralization against other relevant serotypes (BTV-8 and BTV-1) was induced by any of the vaccine prototypes tested in this work, which is consistent with previous results.^58^

No systemic alterations were detected postvaccination. MCT induced a gradual, treatment-related Tbil increase. Previous MCT toxicity studies in animals reported no hepatic effects,^26^ and intravenous tyrosine was well tolerated in sheep.^59^ Most likely, this Tbil increase could be consistent with benign hyperbilirubinemia.^60^ Despite concerns about ApNP toxicity,^61^ the ApNPs used here have shown minimal cytotoxicity,^23^ likely due to their micrometer size when aggregated, that limit them from entering the systemic circulation.^62,63^ These findings reinforce the safety of biomimetic ApNPs as vaccine adjuvants.

The semiquantitative IS reaction analysis used here provides a reliable, noninvasive method for assessing subcutaneous inflammation. IS reactions varied depending on the adjuvant from the most severe reactions induced by AlOOH to the absence of reactions in the BTV-4 and PBS groups. AlOOH-induced reactions after primovaccination were more severe and persistent than those from ApNPs and MCT but were less severe after the booster, while still lasting until the end of the experiment. ApNPs and MCT reactions were more severe after the booster, but these IS reactions resolved between 2 and 3 weeks. The temporal progression of AlOOH-induced reactions was consistent with previously reported intramuscular and subcutaneous responses in mice and sheep, respectively.^7,64^ Local vaccine reactions induced by ApNPs disappeared after 35 days, in contrast to persistent AlOOH granulomas in mice^65^ and MCT-induced lesions resolved after 10 days in both rodent and other mammalian animal models.^26^ Our results indicate that reactogenicity to the injection and immunogenicity induced by the antigen may not be parallel. Reactogenicity and immunogenicity vary between adjuvants and are not always correlated, as has been demonstrated using vaccines against *Mycobacterium avium* subsp. *paratuberculosis*, including different adjuvants.^66^ The reactogenicity-immunogenicity correlation observed with AlOOH and other Al containing adjuvants (such as this study)^67^ may not be generalizable to other adjuvants, and even the role of granulomas in AlOOH-induced protection has been questioned in recent years.^68^ Remarkably, the BTV-4 group produced a profile of antibodies very similar to that of AlOOH, without no apparent reactions, something difficult to explain but of obvious interest in vaccine development. ApNPs, MCT, or even nonadjuvanted BTV vaccines constitute promising safer alternatives than the use of AlOOH.

The AlOOH group was the only showing postinjection granulomas (83%) at post-mortem studies, about four months after vaccine inoculation. None of the other experimental groups demonstrated any reaction at the IS at the end of the experiment, indicating the temporality of the reaction (if any) induced by vaccine prototypes with ApNPs, MCT, or even BTV-4 alone. Al biopersistence in IS reactions has been previously demonstrated in sheep^5^ and in many other species.^1,9^ This persistence is likely linked to Al low solubility in physiological fluids, including acidic phagolysosomes.^5,69^ In the AlOOH group, the differences observed between the prime and booster doses (both in granulomas and ipsilateral lymph nodes) may be attributed to the lower antigen concentration in the booster as the adjuvant concentration remained constant. These aseptic lesions appear to result from AlOOH-antigen interactions, which have been associated with larger necrotic reactions, fewer crystalloid bodies, and increased Al migration to regional lymph nodes, in contrast to granulomas induced by AlOOH only.^5,9^ Inducing an adequate immune response while minimizing long-term adverse reactions is critical for developing safer vaccines. Persistent local reactions, such as those induced by AlOOH, can cause a wide array of clinical signs^70^ and may also even result in carcass trimming.^9^ Moreover, AlOOH has been associated with IS sarcomas in animals,^11,71^ and AlOOH-induced granulomas (such as those induce by BTV vaccines) may serve as sites for replication and amplification of small ruminant lentiviruses, the causative agent of another economically significant disease in sheep.^7^ Both ApNPs and MCT fulfill the criteria to be considered safer adjuvants for future vaccines.

Lymph nodes indicated a similar profile of histopathological changes with an increased reactivity in the AlOOH and MCT groups compared to ApNPs, perhaps indicating a more intensely activation. Macrophage aggregates were seen in all experimental groups, but only aggregates from the AlOOH group demonstrated the presence of Al. Macrophage aggregates were less frequent in ApNP- and MCT-draining lymph nodes, likely reflecting the minimal or absent local lesions observed at necropsy. In contrast, the increased macrophage presence in AlOOH group lymph nodes may indicate enhanced drainage from granulomas.^72^ Our results suggest that Al-laden macrophage migration and local crystalloid body formation may vary with antigen concentration in the prime and booster preparations, with Al migration to lymph nodes inversely correlated with the size and number of crystalloid bodies in granulomas. It is conceivable that a higher antigen load could promote increased activation and migration of macrophages carrying adjuvant nanoparticles, which may partly explain the larger size and volume of the granuloma observed after the prime vaccination. For example, in vitro studies have shown that CCL2 (monocyte chemoattractant protein-1) production, important in macrophage migration, occurs only in the presence of both AlOOH and antigen but not with AlOOH alone.^73^ This might partly account for the differential availability of adjuvant at the IS after prime vaccination, as enhanced migration to the draining lymph node could reduce the amount of AlOOH retained locally for crystalloid body formation—whereas the opposite may have occurred at the booster site. Nonetheless, this remains a speculative interpretation as the present study was not specifically designed to address these mechanisms. Further research is warranted to clarify the potential role of the antigen in modulating adjuvant trafficking rather than focusing solely on the adjuvant itself.

Several experimental limitations should be considered: (1) the relatively small sample size per experimental group, which may limit the statistical power of the analyses; (2) the absence of a direct evaluation of vaccine efficacy through a BTV challenge; (3) the lack of assessment of cell-mediated immune responses, which are critical for a comprehensive immunological profile; (4) the limited follow-up period for monitoring the durability of antibody responses; and (5) the relatively short time frame for overall safety assessment. Additional strategies, such as the repeated application of the vaccination protocol, could help to more thoroughly assess safety in the future.

## 4 Conclusions

Results of this work show compelling evidence suggesting the suitability of ApNPs and MCT as biocompatible, safer, and viable alternative adjuvants for sheep vaccines. Even the use of inactivated BTV alone might be useful as an antigen-inducing immune reaction, without the need of adjuvants. This is a major step in avoiding AlOOH postvaccination reactions in sheep, something that is continuously demanded by veterinarians and farmers. These findings lay the groundwork for future investigations into the applicability of these adjuvants in safer vaccines in sheep, other animal species, humans, as well as against other pathogens.

## 5 Experimental Section

### Animals, Virus, and Adjuvants

Animals in this study were handled in compliance with the Spanish Policy for Animal Protection (RD118/2021) and European Union Directive 2010/63/EU on the protection of experimental animals. The procedures and protocols were approved by the Ethical Committee of the University of Zaragoza (ref. PI34–18). Thirty unvaccinated, 13-month-old, purebred Rasa Aragonesa male sheep (50.04 ± 6.8 kg) were selected from a certified flock free from major sheep diseases and were neutered. A solution of inactivated BTV-4, strain BTV-4/SPA-1/2004, was provided by a veterinary pharmaceutical company. A stock solution of MCT containing MCT in PBS (40 mg mL^–1^) with phenol (0.5% w/v) was supplied by Allergy Therapeutics (Worthing, UK). A stock solution of Adjuval, an AlOOH solution (30 mg mL^–1^) was obtained by CZ Vaccines S.A. (Porriño, Spain). An aliquot of Alhydrogel 2% (Invivogen vac-alu-50), the standard AlOOH adjuvant, was obtained for comparison with Adjuval physicochemical properties. Additionally, biomimetic ApNPs were synthesized following a protocol described elsewhere.^23^ Briefly, two aqueous solutions (1:1 v/v 0.2 L total) were mixed at room temperature: 1) CaCl_2_ (0.1 M), and Na_3_Cit (0.4 M) and 2) Na_2_HPO_4_ (0.12 M) and Na_2_CO_3_ (0.1 M). A white precipitate appeared instantaneously upon mixing. The mixture was then incubated (60 °C for 24 h). Afterward, the precipitate was collected and repeatedly washed with ultrapure water by centrifugation (9000 rpm, 10 min). A hydrogel containing varying concentrations (wt %) of rod-shaped ApNPs was synthesized. PBS at pH 7.4 was purchased from Sigma-Aldrich, Spain.

### Preparation of Inocula

Five inocula were prepared for this experimental study: a PBS solution, BTV-4 alone, and BTV-4 combined with each of the three adjuvants. All inocula were administered as a prime inoculation and subsequently boosted to 21 DPI. The concentration of BTV-4 was not disclosed for confidentiality reasons; however, precise instructions were provided to ensure an equal viral concentration across the three adjuvants used. The prime inoculation contained twice the virus concentration of the booster (Table S14). The relative protein concentration was analyzed using the dye-binding method with a Bio-Rad II protein assay kit (Bio-Rad, USA) and bovine serum albumin (BSA) as the standard, following manufacturer instructions. Data regarding the quantities of adjuvants injected, as well as the ratio of viral protein to adjuvant, is presented in Table S14. For the preparation of the AlOOH inoculum, an appropriate volume of the BTV-4 solution was added to a constantly stirred solution of AlOOH in PBS (6 mg mL^–1^). For the ApNPs inoculum, a hydrogel of these nanoparticles was gradually resuspended in PBS (6.25 mg mL^–1^), followed by 10 min of sonication in an ice bath. The BTV-4 solution was then added with gentle mixing. For the MCT inoculum, the corresponding volume of the BTV-4 solution was added to a constantly stirred solution of MCT in PBS (20 mg mL^–1^). Each inoculum (8 mL) was incubated (24 h at 4 °C) under constant stirring prior to vaccination. A small aliquot of each inoculum was incubated in Brain Heart Infusion (BHI) media at 37 °C, and bacterial growth was assessed at 24, 48, and 72 h.

### Characterization of Adjuvants and Virus

The characterization methodology of adjuvants and virus solution is detailed in Supporting Text 1.

### Inoculation and Sampling Schedule

The study involved 30 lambs and lasted 133 days; the experimental scheme is illustrated in Figure S6. The animals were divided into five groups, each consisting of six animals: the PBS group (inoculated with PBS), the BTV-4 group (inoculated with nonadjuvanted inactivated BTV-4), the AlOOH group (inoculated with AlOOH), the ApNPs group (inoculated with ApNPs adjuvant), and the MCT group (inoculated with MCT adjuvant). Each sheep received a 2 mL subcutaneous prime inoculation in the right flank at 0 DPI, followed by a 2 mL booster inoculation in the left flank at 21 DPI. Blood samples were collected every 21 days, while serum samples were taken weekly. Complete clinical evaluations were conducted at 0 and 28 DPI. ISs were assessed through visualization and palpation (Figure S6). At the conclusion of the experiment, the animals were humanely euthanized, and post-mortem examinations were performed.

### Serological, Hematological, and Biochemical Analysis

Serum samples were analyzed in duplicate using double-recognition ELISA to detect group specific anti-VP7/BTV antibodies (INgezim BTV DR.100.12. BTV.K0, Ingenasa, Spain). The BTV group specific antibody response was measured and graphically represented as the optical density (O.D.) at 450 nm. The negative threshold was set at 15% of the positive control O.D. 450 nm, as indicated by the manufacturer. The presence of homologous neutralizing antibodies against the BTV serotype 4 Morocco strain (MOR2009/09) in vaccinated sheep was assessed using a plaque reduction neutralization test at 35, 70, 119, and 133 DPI, as previously described^74^ and in accordance with the guidelines of the Terrestrial Manual of the World Organization for Animal Health (WOAH).^75^ Briefly, 2-fold dilutions (1:4, 1:8, 1:16, 1:32, 1:64, 1:128) of heat inactivated sheep sera (56 °C for 30 min) were incubated with 100 PFU of BTV-4 (1 h at 37 °C) and then overnight at 4 °C. Subsequently, the samples were inoculated into 96-well plates containing semiconfluent monolayers of Vero cells. After 90 min of virus adsorption, the supernatant was removed, and Dulbecco’s modified eagle medium with fetal calf serum (5%) was added. The plates were incubated for 5 days at 37 °C in CO_2_ (5%). Cells were then fixed and stained with crystal violet (0.3%), formaldehyde (5%), and ethanol (10%). Neutralizing antibody titer was estimated as the highest serum dilution that reduced the cytopathic effect by 50% (PRNT_50_). To determine the presence of cross-neutralizing antibodies, the same procedure was performed using 100 PFU of the heterologous serotypes BTV-1 (ALG2006/01) or BTV-8 (BEL/2006) at 119 DPI. Complete cell blood counts were performed every 21 days at 0, 21, 42, 63, 84, 112, and 126 DPI using an automatic hematological counter IDEXX Procyte Dx (IDEXX laboratories, Westbrook, ME, USA). Serum biochemical examinations were conducted at 0, 7, 28, and 133 DPI using dry biochemistry on the Catalyst One Chemistry Analyzer (IDEXX laboratories, Westbrook, ME, USA). Parameters evaluated are described in Supporting Text 2.

### In Vivo Assessment of Injection Site (IS) Reactions

Palpation of the prime inoculation and booster ISs was performed on the days indicated in Figure S6. ISs were shaved prior to each vaccination to optimize the evaluation. To assess the magnitude and persistence of the *in vivo* local reaction between groups, a semiquantitative scale based on four grades was applied: grade 0 or not found (not detected by palpation), grade 1 or small (mild bump; could not be retained between thumb and forefinger), grade 2 or medium (moderate bump; could be retained between thumb and forefinger with difficulty), and grade 3 or large (severe bump; could be easily retained between thumb and forefinger). Corresponding results were expressed as relative frequencies of each grade of IS per day of evaluation.

### Pathological Studies

Post-mortem studies were conducted on all animals at the end of the experiment, which included the location and sampling of ISs. Samples for histopathological analysis were obtained from the ISs (*n* = 60), right and left axillary lymph nodes, prescapular lymph nodes, and internal organs. Tissues were fixed in 10% neutral-buffered formalin and embedded in paraffin wax, and 4 μm sections were cut for HE staining. In addition, the MAH stain, a specific technique for Al detection in tissues, was performed on the ISs (*n* = 10), on the right and left axillary (*n* = 11) and prescapular lymph nodes (*n* = 12) of the AlOOH group, following a previously published methodology.^6^ MAH results were contrasted with lumogallion fluorescence staining.^5^ Gram, Ziehl-Nielsen, and Grocott-Gomori methenamine silver stains were performed on ISs from the AlOOH group to rule out the presence of bacteria, acid-fast organisms, or fungi, respectively. Alizarin Red and Von Kossa staining were used to detect calcium deposits in lymph nodes from ApNPs-treated animals with macrophage aggregates (*n* = 3), while birefringence was evaluated in lymph nodes from the MCT group. Histological features indicative of hyperplasia in draining lymph nodes (prescapular and axillary) were assessed by two European board-certified veterinary pathologists (ECVP) according to a semiquantitative scoring system (Table S15, Figures S7, S8, and S9). Evaluation was designed for different compartments following the Society of Toxicologic Pathology guidelines.^76^ Analyzed compartments included CPT, SF, MP, MH, and FH as follicular hyaline lakes (Figure S5B) were also scored. In an independent analysis, macrophage aggregates, defined as clusters of ≥4 macrophages with foamy to granular cytoplasm (Figure S5A), were evaluated as described elsewhere.^5^ A study on the crystalloid bodies present in AlOOH granulomas was performed by MAH in ISs, determining counts at 10× and length, width, and aspect ratio (AR; the ratio between the longest diameter of a particle to the shortest perpendicular diameter) at ×40. An index was calculated in both ISs and lymph nodes by dividing the counts of crystalloid bodies or the counts of macrophage aggregates by the number of fields studied in each case.

### Statistical Analysis

All statistical analyses were performed using IBM SPSS 19.0 for Windows (IBM Corp., Armonk, NY, USA), and graphs were generated with OriginPro (OriginPro 9.8.0, OriginLab Corporation, USA). Z potential data were analyzed by repeated measures ANOVA followed by Duncan’s Multiple Range (DMR) test. Laser diffraction data were studied by One-Way ANOVA followed by DMR tests. Group specific antibodies were analyzed using repeated measures ANOVA, followed by posthoc comparisons with Dunnett’s or DMR test, as appropriate. At each time point, groups were compared using Duncan’s test. Dunnett’s test was applied to compare each group’s weekly values with those of all animals at 0 DPI (EB; *n* = 30) and to assess the booster effect. Neutralizing antibody titers were analyzed using a Kruskal–Wallis (KW) test and a Mann–Whitney U (MW-U) test. Comparisons between DPI among groups were analyzed using Friedman and Wilcoxon signed-rank (WSR) tests. Circulating monocyte levels were analyzed using factorial between-subjects ANOVA, with posthoc comparisons conducted using DMR test. Comparisons with 0 DPI were performed using Student’s *t* test or WSR tests. Tbil data were analyzed using KW and MW-U tests for comparison among groups and the Friedman and WSR tests for comparisons among DPI. Stratified frequencies were described using contingency tables for the analysis of ISs, lymphoid hyperplasia, and macrophage aggregates in lymph nodes. The chi-square (χ^2^) test was used to compare observed data with the expected frequency assuming conditional independence between groups and IS reactions. Associations between variables were assessed with the χ^2^ test when less than 20% of cells had an expected value below 5 and with the likelihood-ratio (LR) test otherwise. Expected frequencies were established under the assumption of conditional independence at a 95% confidence level. Adjusted standardized residuals (ASR) were then analyzed to detect significant deviations in categorical variable associations. Interpretation thresholds followed standard normal distribution critical values: ±1.96 (*p* > 0.05) = Nonsignificant deviation; ±1.96 to ± 2.58 (0.01 < *p* < 0.05) = Marginal significance and ±2.58 to ± 3.29 (0.001 < *p* < 0.01): Strong evidence against independence. The total score for all evaluated changes of lymphoid hyperplasia was analyzed using intersubject ANOVA followed by DMR test. A stepwise forward binomial logistic regression was used to estimate significant associated variables with the presence/absence of macrophage aggregates in lymph nodes, calculating the Odds Ratios as the exponent of beta coefficients. Crystalloid bodies count in AlOOH ISs were analyzed using independent-samples Student’s *t* test for comparison between the right and left side and paired *t* test for comparisons within subjects. Characteristics of Al crystalloid bodies (length, width, and AR) in the right (prime inoculation) and left ISs (booster) were compared using the MW-U test. Lymph node Al-laden macrophage migration indices were similarly compared using the MW-U test for independent samples and the WSR test for intraindividual dependence. Key data on antibody levels, Al-laden macrophage migration, crystalloid body sizes, and hematology were presented as median ± interquartile range for non-normal distributions. Crystalloid body counts and the total score of lymphoid hyperplasia analysis were reported as mean ± SD, while physicochemical characterization data were expressed as mean ± standard error of the mean with 95% confidence intervals (95% CI). Statistical significance was set at *p* < 0.05.

### Safety Considerations

No unexpected, new, or significant hazards or risks were associated with the reported work.

## Supplementary Material



## Data Availability

The data that support the findings of this study are available within the article and its Supporting Information.

## Notes

XRD patterns of adjuvants (Adjuval, Alhydrogel, MCT, and ApNPs); viral cross-neutralization results for BTV-1 and BTV-8; blood monocyte levels by group (total and by DPI); Tbil levels by group and DPI; the only in vivo injection site (IS) reaction observed in one animal vaccinated with ApNPs; post-mortem findings at the IS of MCT; histopathological images of macrophage aggregates in lymph nodes considered and evaluated; histopathological image of follicular hyalinosis (FH) in regional lymph nodes; experimental timeline of vaccination and sampling; histopathological images illustrating scoring criteria for cortex–paracortex thickening (CPT), secondary follicles (SF), medullary plasmacytosis (MP), and medullary histiocytosis (MH) in lymph nodes; table of physicochemical characteristics of BTV-4, adjuvants, and vaccine formulations; table of group-specific anti-BTV antibody levels and between-group comparisons by date; table of the effects of prime and booster inoculations on group-specific anti-BTV antibody levels; table comparing neutralizing antibody titers against BTV-4 between groups by date; table of temporal comparisons of neutralizing antibody titers against BTV-4 within each group; table of red blood cell parameters per group; table of white blood cell and platelet counts per group; table of biochemical parameters per group; data from exploratory clinical analyses per group; table of relative frequencies and statistical analysis by grade, group, and date for ISs following prime inoculation; table of relative frequencies and statistical analysis by grade, group, and date for ISs following booster inoculation; table of comparative analysis of CPT, SF, MP, MH, and FH by group; table of comparative analysis of macrophage aggregates (Mφ) by group; table of adjuvant quantities administered per dose, animal, and weight; table of microscopic criteria and scoring for lymphoid hyperplasia changes in prescapular and axillary lymph nodes; experimental section detailing adjuvant, virus, and formulation characterization; and experimental section describing complete blood counts and clinical chemistry (PDF)

## ●

Universitat Autònoma de Barcelona, Bellaterra, Barcelona 08193, Spain

## ▲

AnaPath Services GmbH, 49 Hammerstrasse Street, Liestal 4410, Switzerland.

## ABBREVIATIONS

AlOOH: aluminum oxyhydroxide
Al: aluminum
BTV: bluetongue virus
ApNPs: apatite nanoparticles
MCT: microcrystalline tyrosine
IS: injection site
TEM: transmission electron microscopy; HAADF-STEM, high-angle annular dark-field scanning transmission electron microscopy
LDA: laser diffraction analysis
XRD: X-ray diffraction
DPI: days postprimovaccination
EB: experimental baseline
PRNT: plaque reduction neutralization test
Tbil: total bilirubin
PBS: phosphate buffered saline
MAH: modified-aluminum hematoxylin
CPT: cortex-paracortex thickening
SF: secondary follicles
MP: medullary plasmacytosis
MH: medullary histiocytosis
FH: follicular hyalinosis
LFN: lymph node
KW: Kruskal Wallis
DMR: Duncan’s multiple range test
MW: Mann–Whitney U test; WSR, Wilcoxon signed-rank test
