# Supplementary material for: Biomimetic Apatite Nanoparticles and Microcrystalline Tyrosine as Biocompatible Vaccine Adjuvants: Performance in a Bluetongue Virus Sheep Model
Source: ACS Appl Mater Interfaces. 2025 Jul 8;17(32):45538–54. doi: 10.1021/acsami.5c10402 (PMC12356533; doi:10.1021/acsami.5c10402)
Supplement: Supplementary file 1 [file am5c10402_si_001.pdf]

Supporting information

# Biomimetic Apatite Nanoparticles and Microcrystalline Tyrosine As Biocompatible Vaccine Adjuvants: Performance In a Bluetongue Virus Sheep Model

*Estela Pérez [a], Víctor Sebastián [bcd], Ana Rodríguez-Largo [a#], Ricardo de Miguel [a$], Álex Gómez[ae], Matthias F. Kramer[f], Anke Graessel [f], Belén Parra-Torrejón [g], José Manuel Delgado-López [g], Sergio Utrilla-Trigo [h], Luis Jiménez-Cabello [h], Javier Ortego [h], Ignacio de Blas [e], Ramsés Reina [i], Marta Pérez [ej], Lluís Luján [ae*]*

[a] Department of Animal Pathology, University of Zaragoza, 177 Miguel Servet Street, Zaragoza, 50013, Spain.

[b] Institute of Nanoscience and Materials of Aragon (INMA), CSIC-University of Zaragoza, Mariano Esquillor Gómez Street I+D+i building, Zaragoza, 50018, Spain.

[c] Department of Chemical and Environmental Engineering, University of Zaragoza, María de Luna 3 Street, Zaragoza, 50018, Spain.

[d] Advanced Microscopy Laboratory, University of Zaragoza, Mariano Esquillor Gómez Street I+D+i building, Zaragoza, 50018, Spain.

[e] Agri-Food Institute of Aragon (IA2), University of Zaragoza, 177 Miguel Servet Street, Zaragoza, 50013, Spain.

[f] Bencard Adjuvant Systems, Allergy Therapeutics PLC, Dominion Way Street, Worthing, BN14 8SA, UK.

[g] Department of Inorganic Chemistry, University of Granada, Fuentenueva avenue, Granada, 18071, Spain.

[h] Center of research in animal health (CISA-INIA, CSIC), Algete-El Casar road, km. 8.1, Madrid, 28130, Spain.

[i] Institute of Agrobiotechnology, CSIC-Government of Navarra, Avenue of Pamplona, Mutilva, 31192, Spain.

[j] Department of Anatomy, Embryology and Genetics, University of Zaragoza, 177 Miguel Servet Street, 50013, Spain.

*E-mail: Lluis.Lujan@unizar.es

# Supporting information index

***1. Supporting Figures*** ***3***
Figure S1. 3
Figure S2. 4
Figure S3. 5
Figure S4. 6
Figure S5. 7
Figure S6. 8
Figure S7. 9
Figure S8. 10
Figure S9. 10
***1.2. Supporting Tables*** ***11***
Table S1. 11
Table S2. 12
Table S3. 13
Table S4. 14
Table S5. 15
Table S6. 16
Table S7. 17
Table S8. 18
Table S8. 19
Table S9. 20
Table S10. 21
Table S11. 22
Table S12. 23
Table S13. 25
Table S14. 26
Table S15. 27
***1.3. Supporting Texts*** ***28***
1.3.1. Supporting Text 1. Adjuvants and viral characterization 28
1.3.2. Supporting Text 2. Complete cell blood counts and clinical chemistry 29
***1.4. References of supporting information*** ***30***

## 1. Supporting Figures

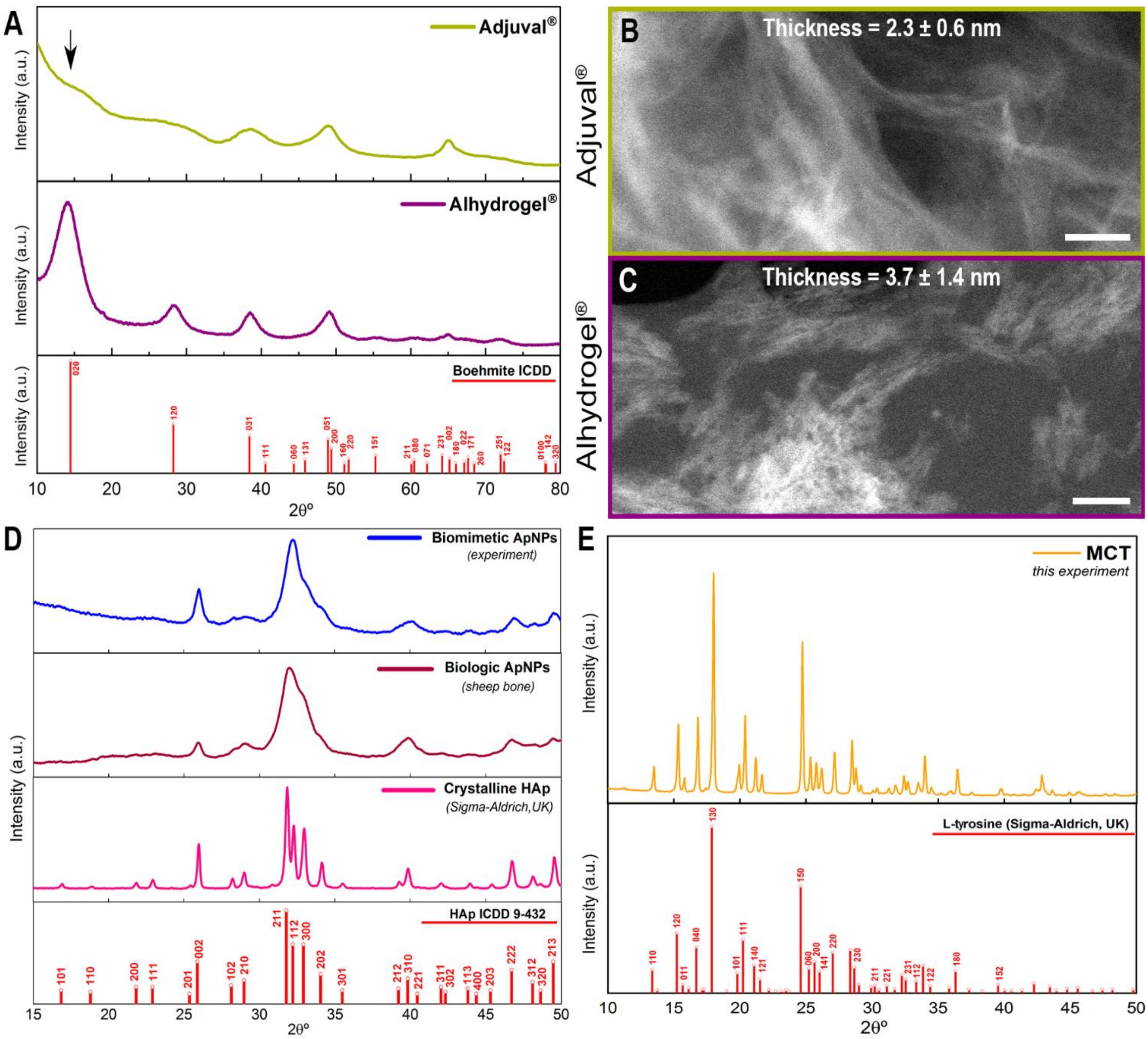


**Figure S1.** Adjuvant XRD patterns. **A.** Adjuval® and Alhydrogel® were compatible with pseudoboehmite (ICDD No. 01-072-03559 - γAlOOH). Broadened peaks in Adjuval®, especially at diffraction plane 020 (arrow), suggest a thinner particle size and lower crystallinity [1,2]. **B-C.** Particle morphology and thickness for Adjuval® (scale bar: 20 nm) (B) and Alhydrogel (Scale bar: 50 nm) (C). **D.** Biomimetic apatite nanoparticles (ApNPs) were identified as a single-phase hydroxyapatite (HAp) (ICDD 9-432), exhibiting poor crystallinity compared to mineral HAp (Sigma-Aldrich, UK) and displaying characteristics identical to those of a sheep bone sample. **E.** MCT® displays a highly crystalline structure corresponding to L-tyrosine (Sigma-Aldrich, UK), consistent with prior characterization (Shardlow and Exley, 2019).

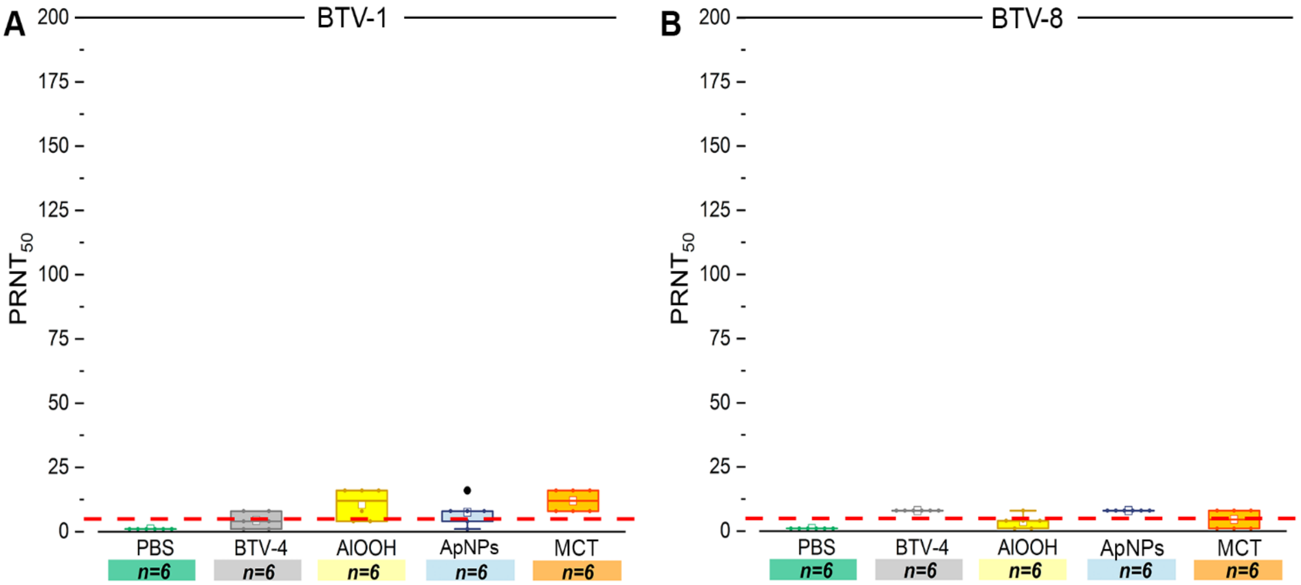


**Figure S2.** Viral cross-neutralization assay for serotypes BTV-1 (A) and BTV-8 (B) at 119 DPI. No neutralizing titers are detected. BTV-1, BTV-8; serotypes 1 and 8 of bluetongue virus. $PRNT_{50}$; highest serum dilution at which the cytopathic effect of the virus was reduced by 50%. Red dash line: cut-off (1:5).

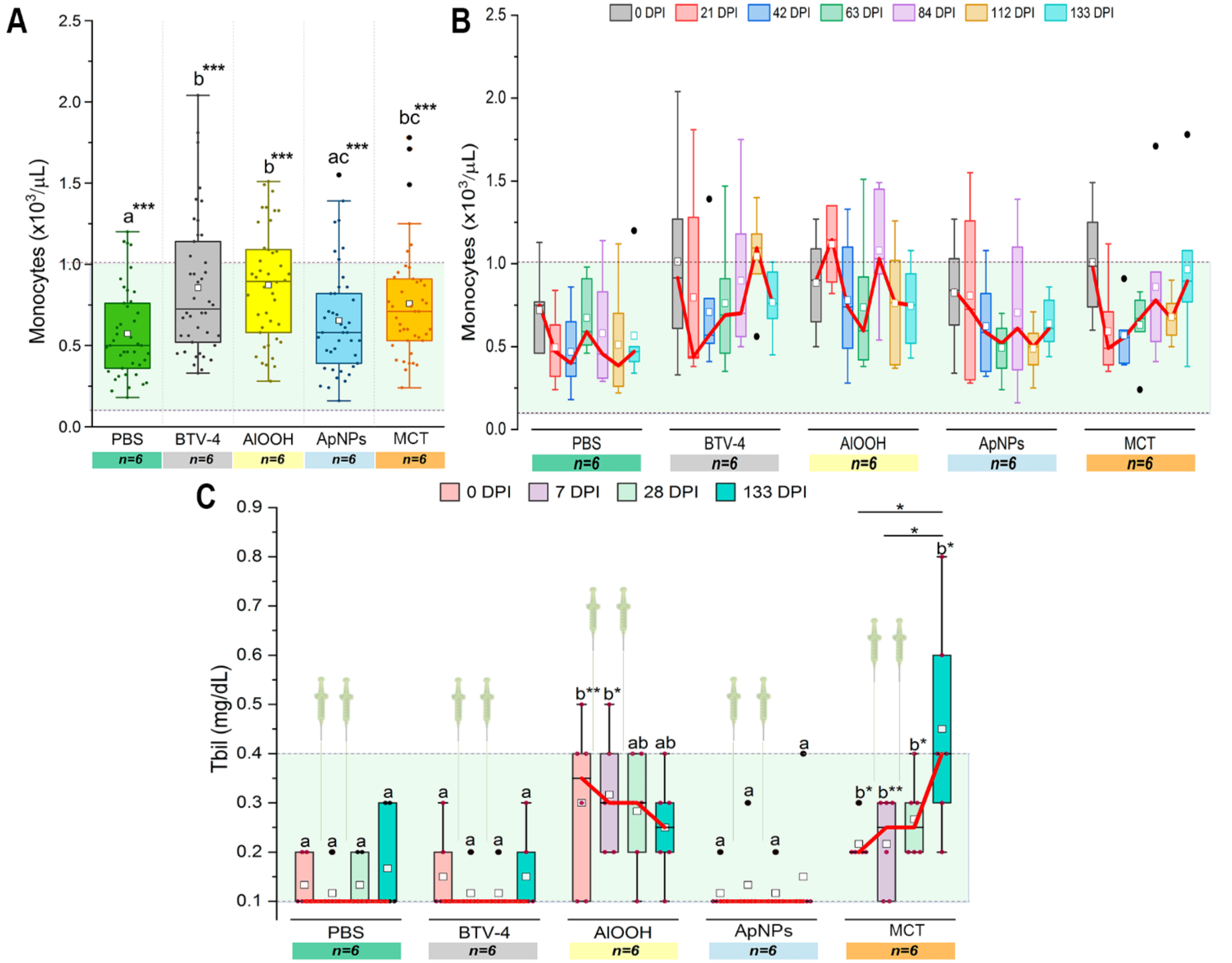


**Figure S3. (A-B)**. Blood cell counts. **(A)** Total blood monocyte levels by treatment group; **(B)** Temporal evolution of monocytes by treatment group; and **(C)** Clinical chemistry. Temporal evolution of Tbil levels by treatment group. Boxplots represent the interquartile ranges for each group, with the mean indicated by a central dot and the median by horizontal lines. Whisker indicates minimum and maximum values, excluding outliers (black dots). Medians are connected by a red line in B and C. The general reference range of the Catalyst One Chemistry Analyzer (IDEXX laboratories, USA) is depicted as a horizontal green band. **A.** Different vowels denote significant differences. **B.** Significant deviations from baseline are indicated for MCT group. **C.** Different vowels denote significant differences. $*p < 0.05$; $**p < 0.01$; DPI, days post prime inoculation.

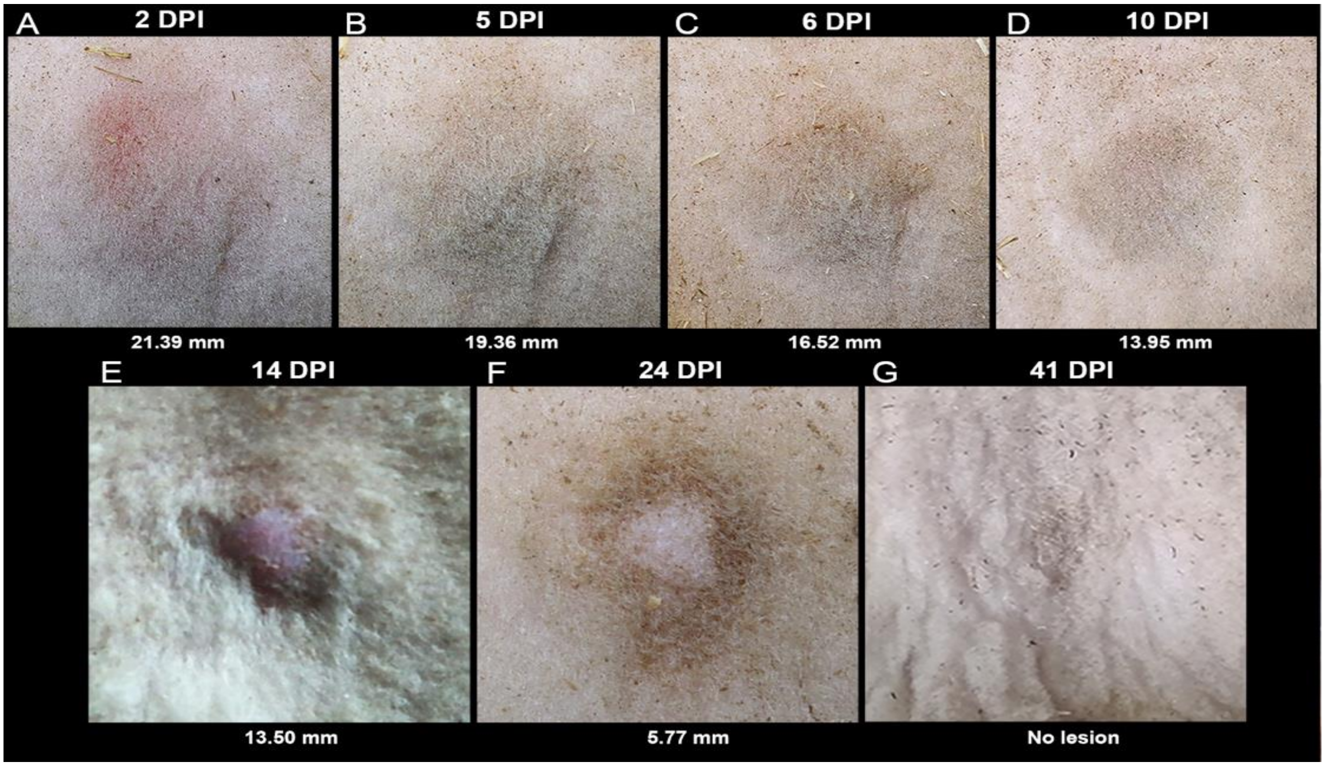


**Figure S4. A-G:** ApNPs booster inoculation. Evolution of the lesions detected at the injection site (IS) in a single animal. The acute inflammation observed at 48 hours (2 DPI) **(A)** fully resolves by 41 days post-booster inoculation **(G)**, the most rapid resolution occurring within the first 10 days **(A-D)**.

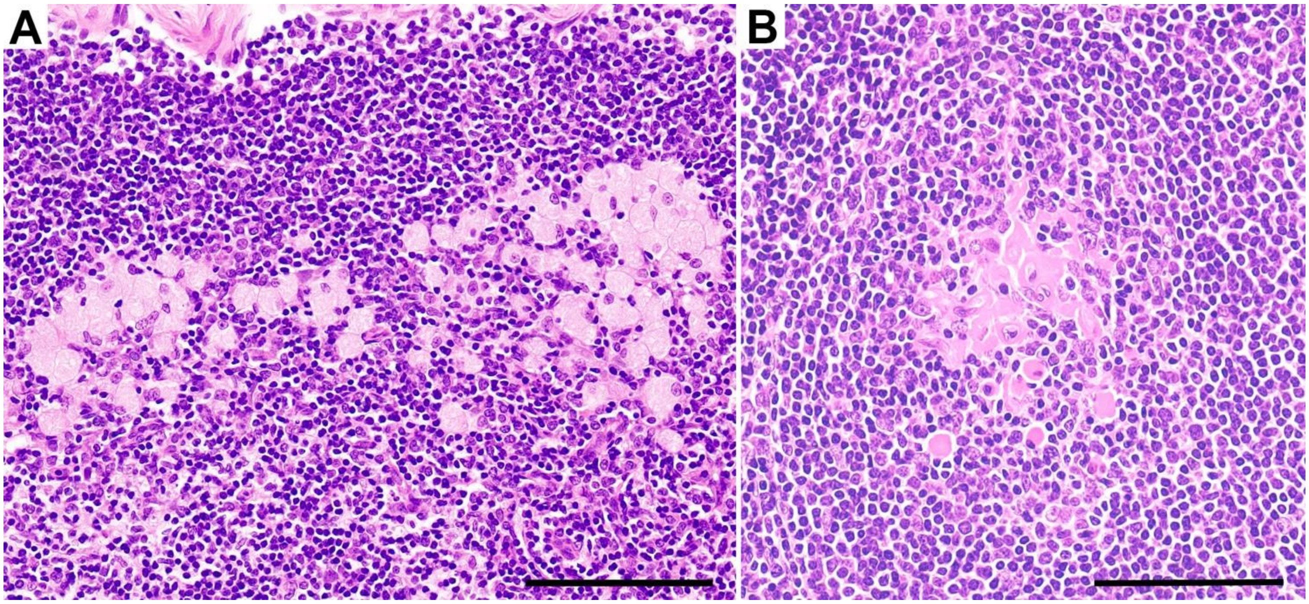


**Figure S5. A.** Regional lymph node. Presence evaluation of conspicuous foamy to granular cytoplasm macrophages. HE, bar: 100 µm. **B.** Follicular hyalinosis score was determined by the number of germinal centers exhibiting central lacunae of hyaline material, as shown. HE, bar: 50 µm.

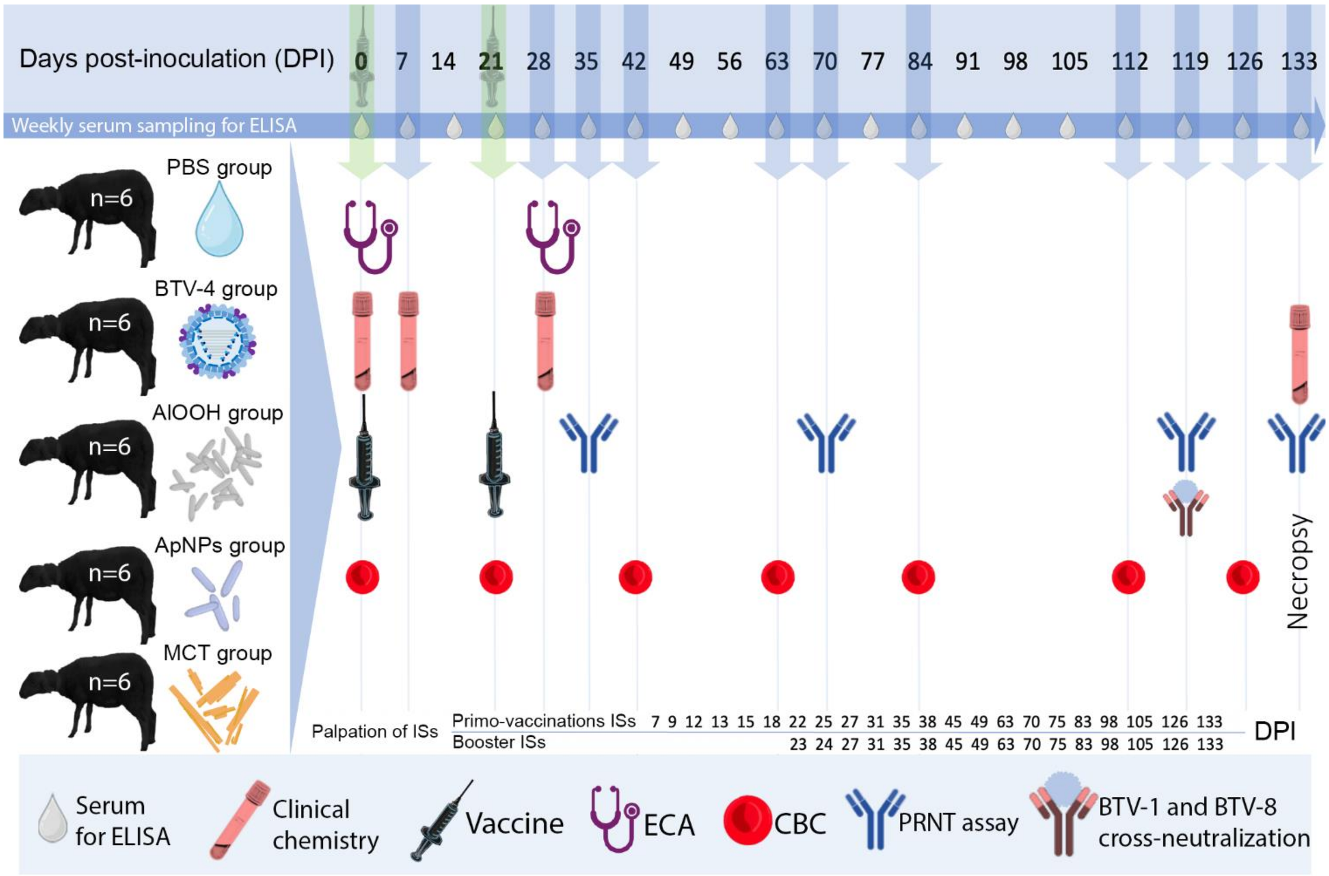


**Figure S6.** Experimental timeline of vaccination and sampling. *PBS group*, vaccinated with phosphate buffer saline only; *BTV-4 group*, vaccinated with non-adjuvanted inactivated bluetongue virus serotype 4; *AlOOH group*, vaccinated with inactivated virus adjuvanted with aluminum oxyhydroxide, *ApNPs group*, vaccinated with inactivated virus adjuvanted with biomimetic apatite nanoparticles, *MCT group*, vaccinated with inactivated virus adjuvanted with MCT; ISs; injection sites; ECA, exploratory clinical analysis; CBC, complete blood count; PRNT; Plaque reduction neutralization test; BTV-1, BTV-8, BTV-4; Bluetongue virus serotypes 1, 8 and 4.

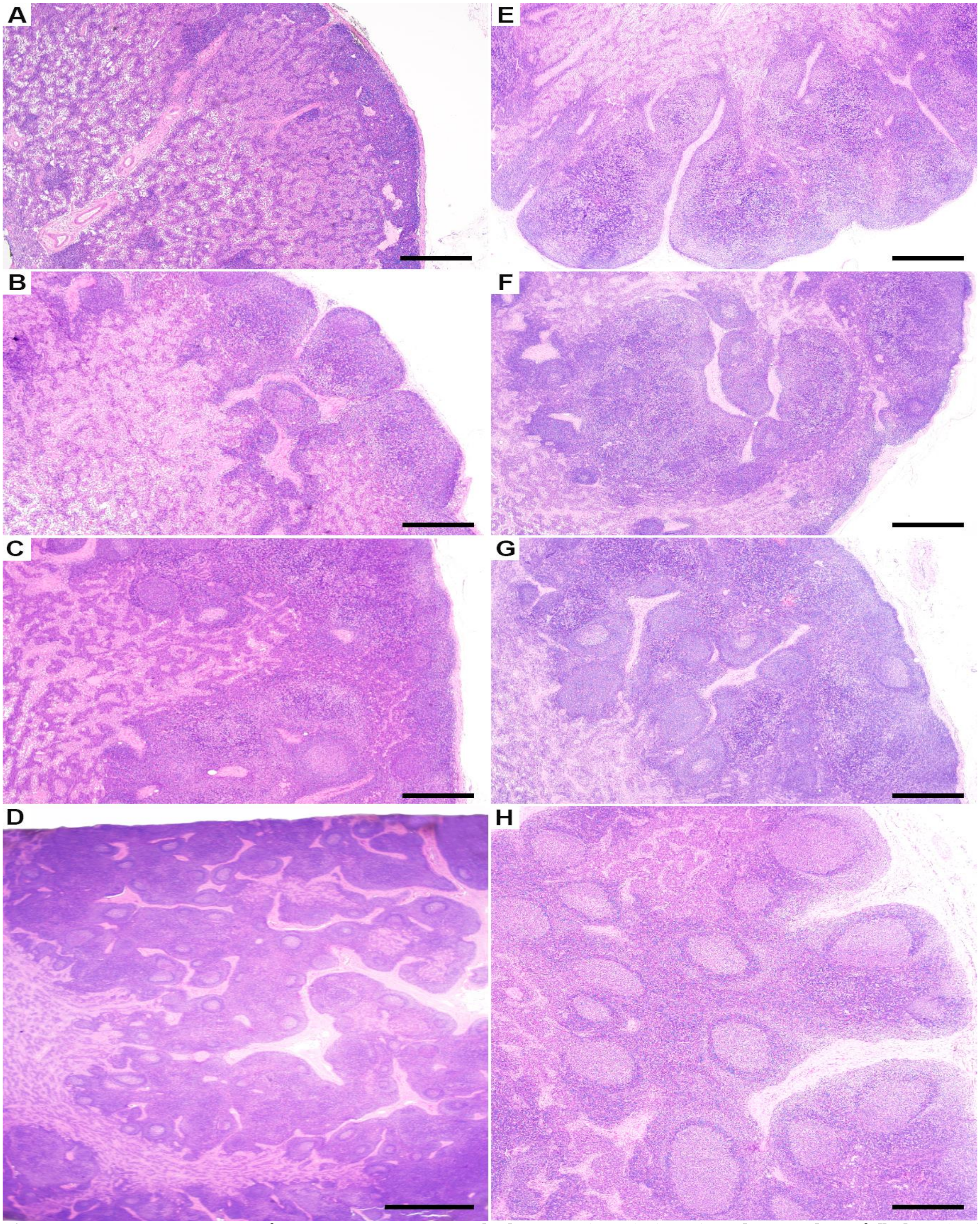


**Figure S7.** Scoring criteria for cortex-paracortex thickening (CPT) (A-D) and secondary follicles (SF) (E-H). **A-D.** CH. **A, E.** Score 0. **B,F.** Score 1. **C, G.** Score 2. **D, H**. Score 3. **A-H.** HE, bars: 300 µm.

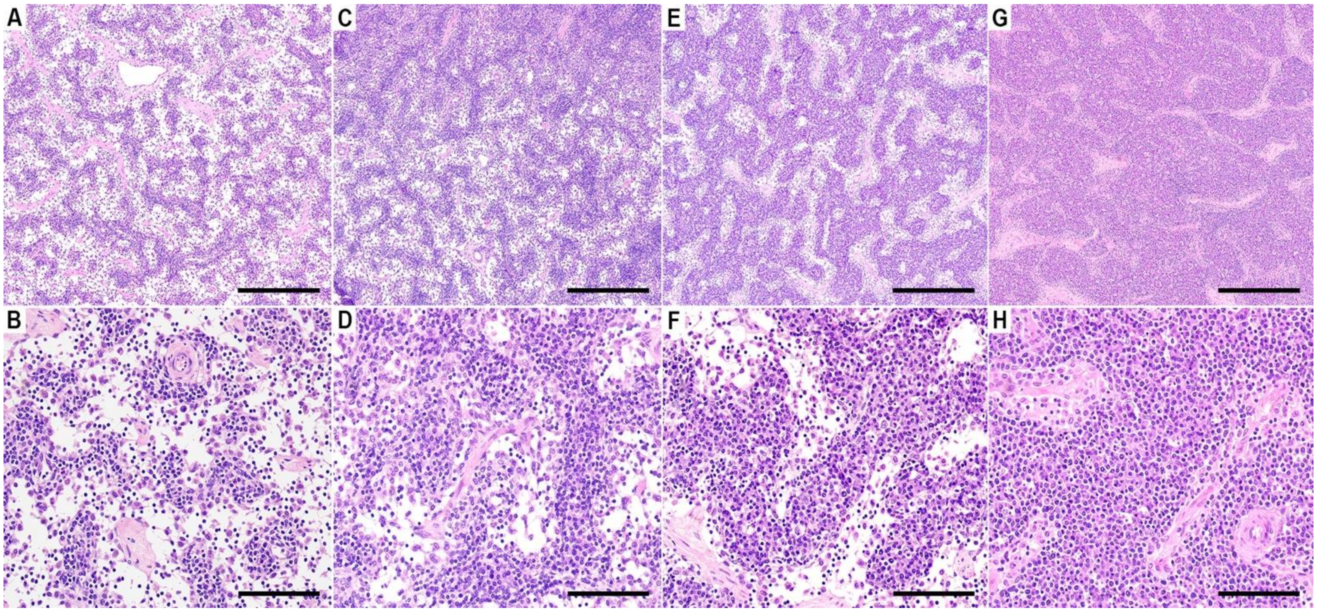


**Figure S8.** Scoring criteria for medullary plasmacytosis (MP) (A-H). **A-B.** Score 0. **C-D.** Score 1. **E-F.** Score 2. **G-H.** Score 3. A,C, E, G. HE, bar: 200 µm. B, D, F, H. HE, bar: 50 µm.

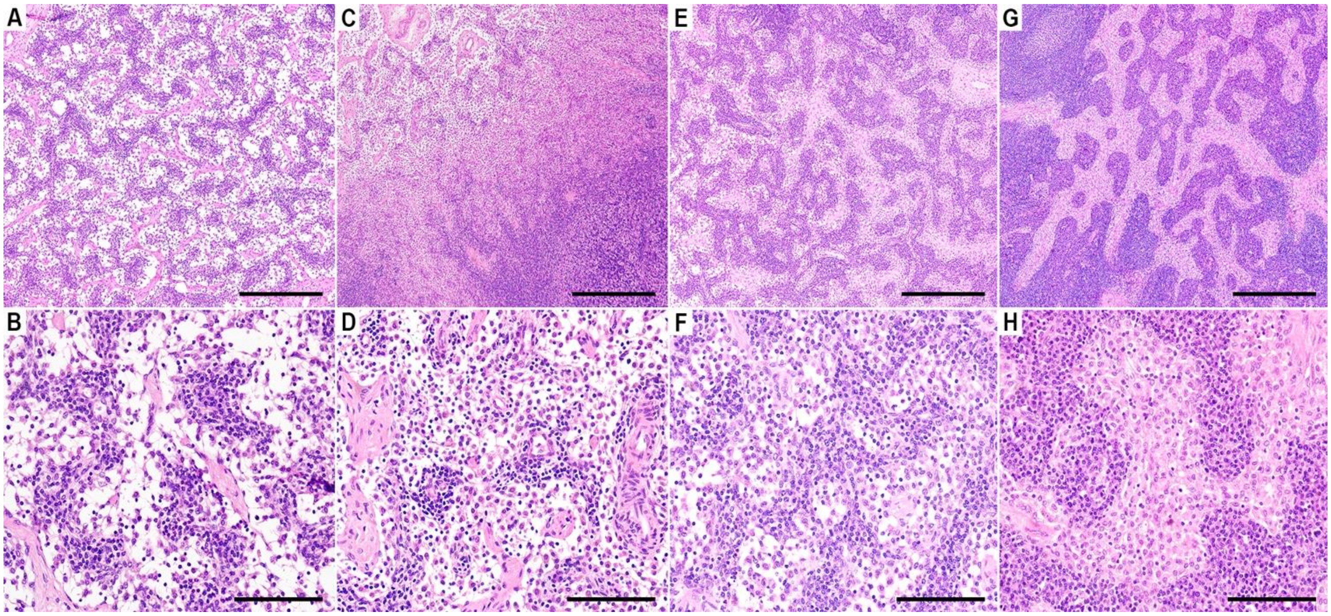


**Figure S9.** Scoring criteria for medullary histiocytosis (MH) (A-H). **A-B.** Score 0. **C-D.** Score 1. Only a band at the interface with the paracortex. **E-F.** Score 2. **G-H.** Score 3. A,C, E, G. HE, bar: 200 µm. B, D, F, H. HE, bar: 50 µm.

## 1.2. Supporting Tables

**Table S1.** Physicochemical characteristics studied on BTV-4, adjuvants and formulations used in this study.

| Sample | ζ **Potential** ***mV*** [a] | **D[4,3] μm**[b] | **Dv10 μm**[b] | **Dv50 μm**[b] | **Dv90 μm**[b] | **Span**[b] |
|---|---|---|---|---|---|---|
| | | | *M ±SEM [95% CI]* | | | |
| BTV-4 | ***(b)***11.2 ± 0.6 [-12.4, -9.9] | n/d | n/d | n/d | n/d | n/d |
| | | ***(d)****M1*: 9.8 ± 0.4 [4.9; 14.7] | ***(c)****M1*: 4.7 ± 0.0 [4.4; 5.0] | ***(d)****M1:* 8.9 ± 0.3 [5.4; 12.5] | ***(f)****M1:* 16.7 ± 1.0 [4.6; 28.9] | ***(fe)****M1:* 1.4 ± 0.1 [0.5; 2.2] |
| AlOOH | ***(b)***-11.4 ± 0.5 [-12.30, -10.4] | ***(bc)****M2*: 37.5 ± 5.2 [-28.5; 103.5] | ***(b)****M2*: 22.4 ± 1.6 [1.7; 43.0] | ***(cb)****M2:* 34.4 ± 4.0 [-16.6; 85.3] | ***(cde)****M2:* 58.4 ± 11.5 [-87.5; 204.2] | ***(f)****M2:* 1.0 ± 0.2 [-1.1; 3.2] |
| | | ***(a)****M3*: 204.6 ± 33.1 [-216.0; 625.2] | ***(a)****M3*: 128.6 ± 26.8 [-212.5; 469.7] | ***(a)****M3*: 194.8 ± 34.4 [-241.6; 631.2] | ***(a)****M3:* 296.7 ± 38.0 [-185.6; 779.0] | ***(f)****M3*: 0.9 ± 0.1 [-0.4; 2.2] |
| ApNPs | ***(a)***-18.0 ± 0.6 [-19.2, -16.7] | ***(cd)***18.2 ± 0.7 [15.3; 21.0] | ***(cb)***6.5 ± 0.3 [5.2; 7.9] | ***(cd)***14.4 ± 0.6 [11.7; 17.0] | ***(ef)***32.8 ± 1.4 [26.6; 39.0] | ***(ed)***1.8 ± 0.0 [1.8; 1.9] |
| MCT | ***(b)***-10.3 ± 0.6 [-11.53, -9.02] | ***(bcd)***23.3 ± 0.3 [22.2; 24.4] | ***(cb)***5.9 ± 0.1 [5.4; 6.4] | ***(bcd)***15.4 ± 0.2 [14.5; 16.2] | ***(cde)***51.6 ± 0.4 [49.9; 53.3] | ***(ab)***3.0 ± 0.0 [2.9; 3.1] |
| AlOOH/ BTV-4 | n/d | ***(b)***41.3 ± 3.0 [28.5; 54.1] | ***(cb)***15.0 ± 1.5 [8.7; 21.3] | ***(b)***36.9 ± 3.2 [23.4; 50.5] | ***(b)***90.6 ± 7.6 [57.9; 123.3] | ***(cde)***2.1 ± 0.3 [1.0; 3.2] |
| ApNPs/ BTV-4 | n/d | ***(bc)***33.0 ± 3.2 [19.5; 46.6] | ***(cb)***7.7 ± 0.1 [7.2; 8.3] | ***(bcd)***21.3 ± 1.1 [16.6; 26.0] | ***(bcd)***72.4 ± 6.4 [44.8; 100.1] | ***(a)***3.0 ± 0.2 [2.3; 3.7] |
| MCT/ BTV-4 | n/d | ***(bcd)***22.7 ± 1.4 [16.7; 28.7] | ***(cb)***5.7 ± 0.5 [3.7; 7.8] | ***(bcd)***14.7 ± 0.2 [13.8; 15.7] | ***(cde)***50.0 ± 5.6 [25.7; 74.3] | ***(ab)***3.0 ± 0.4 [1.2; 4.9] |

*D[4,3]*, volume-weighted mean diameter; *Dv10, Dv50, Dv90*; 10%, 50% (median) and 90% of the cumulative volume distribution; *M*, mean; *SEM*, standard error of the mean; *CI*, confidence interval of the mean [upper limit, lower limit]; *n/d*, not determined; *M1, M2, M3*, mode 1, 2 and 3; [a]Different letters indicate significant differences ($p = 0.01$); Duncan's Multiple Range (DMR) test; [b]Different letters indicate significant differences ($p < 0.001$); DMR test.

**Table S2.** Group specific antibody levels against BTV and comparison between groups by date.

| DPI | Group | | | | | $p_{RMA}$ |
|---|---|---|---|---|---|---|
| | **PBS** *n = 6* Md ± IQR[a] *[O.D.450 nm]* | **BTV-4** *n = 6* Md ± IQR[a] *[O.D.450 nm]* | **AlOOH** *n = 6* Md ± IQR[a] *[O.D.450 nm]* | **ApNPs** *n = 6* Md ± IQR[a] *[O.D.450 nm]* | **MCT** *n = 6* Md ± IQR[a] *[O.D.450 nm]* | |
| ***0*** | 0.06 ± 0.02-**a** | 0.08 ± 0.02-**a** | 0.06 ± 0.01-**a** | 0.06 ± 0.19-**a** | 0.08 ± 0.01-**a** | 0.166 |
| ***7*** | 0.05 ± 0.01-**a** | 1.44 ± 0.37-**d** | 1.30 ± 0.40-**cd** | 1.19 ± 0.67-**c** | 0.53 ± 0.12-**b** | **<0.001 ***** |
| ***14*** | 0.04 ± 0.04-**a** | 0.87 ± 0.39-**c** | 1.07 ± 0.37-**c** | 1.12 ± 0.69-**c** | 0.39 ± 0.21-**b** | **<0.001 ***** |
| ***21*** | 0.04 ± 0.02-**a** | 0.40 ± 0.27-**b** | 0.68 ± 0.30-**b** | 0.70 ± 0.70-**d** | 0.20 ± 0.12-**ab** | **0.001 **** |
| ***28*** | 0.04 ± 0.01-**a** | 0.52 ± 0.39-**bc** | 0.34 ± 0.23-**ab** | 1.06 ± 0.37-**d** | 0.72 ± 0.36-**cd** | **<0.001 ***** |
| ***35*** | 0.04 ± 0.01-**a** | 0.48 ± 0.42-**bc** | 0.29 ± 0.14-**ab** | 1.03 ± 0.24-**d** | 0.74 ± 0.57-**c** | **<0.001 ***** |
| ***42*** | 0.04 ± 0.01-**a** | 0.53 ± 0.38-**b** | 0.38 ± 0.19-**b** | 0.99 ± 0.30-**c** | 0.74 ± 0.61-**b** | **<0.001 ***** |
| ***49*** | 0.05 ± 0.00-**a** | 0.60 ± 0.34-**b** | 0.60 ± 0.63-**b** | 1.06 ± 0.37-**b** | 0.74 ± 0.72-**b** | **<0.001 ***** |
| ***56*** | 0.05 ± 0.00-**a** | 0.67 ± 0.28-**b** | 0.60 ± 0.62-**b** | 1.00 ± 0.35-**b** | 0.75 ± 0.73-**b** | **<0.001 ***** |
| ***63*** | 0.05 ± 0.00-**a** | 0.78 ± 0.27-**b** | 0.57 ± 0.62-**b** | 1.03 ± 0.24-**b** | 0.70 ± 0.66-**b** | **<0.001 ***** |
| ***70*** | 0.05 ± 0.00-**a** | 0.97 ± 0.25-**b** | 0.56 ± 0.31-**b** | 1.13 ± 0.35-**b** | 0.95 ± 0.71-**b** | **<0.001 ***** |
| ***77*** | 0.05 ± 0.01-**a** | 1.09 ± 0.55-**b** | 0.65 ± 0.46-**b** | 1.16 ± 0.50-**b** | 0.97 ± 0.74-**b** | **<0.001 ***** |
| ***84*** | 0.05 ± 0.01-**a** | 1.23 ± 0.45-**b** | 0.79 ± 0.46-**b** | 1.22 ± 0.48-**b** | 0.98 ± 0.67-**b** | **<0.001 ***** |
| ***91*** | 0.05 ± 0.02-**a** | 1.16 ± 0.39-**c** | 0.93 ± 0.87-**c** | 1.44 ± 0.35-**c** | 0.91 ± 0.76-**b** | **<0.001 ***** |
| ***98*** | 0.06 ± 0.01-**a** | 1.25 ± 0.18-**c** | 1.00 ± 0.86-**c** | 1.45 ± 0.37-**c** | 0.94 ± 0.60-**b** | **<0.001 ***** |
| ***105*** | 0.05 ± 0.01-**a** | 1.32 ± 0.12-**c** | 1.12 ± 0.77-**c** | 1.42 ± 0.28-**c** | 0.87 ± 0.45-**b** | **<0.001 ***** |
| ***112*** | 0.05 ± 0.01-**a** | 1.41 ± 0.18-**c** | 1.21 ± 0.60-**c** | 1.51 ± 0.37-**c** | 1.04 ± 0.17-**b** | **<0.001 ***** |
| ***119*** | 0.06 ± 0.02-**a** | 1.47 ± 0.22-**c** | 1.27 ± 0.60-**bc** | 1.49 ± 0.31-**b** | 1.08 ± 0.16-**c** | **<0.001 ***** |
| ***126*** | 0.06 ± 0.01-**a** | 1.47 ± 0.12-**bc** | 1.31 ± 0.54-**bc** | 1.60 ± 0.19-**c** | 1.19 ± 0.34-**b** | **<0.001 ***** |
| ***133*** | 0.05 ± 0.01-**a** | 1.48 ± 0.23-**b** | 1.32 ± 0.52-**b** | 1.89 ± 0.16-**c** | 1.19 ± 0.32-**b** | **<0.001 ***** |
| ***Total*** | 0.49 ± 0.01-**a** | 1.06 ± 0.77-**d** | 0.80 ± 0.79-**c** | 1.17 ± 0.59-**e** | 0.86 ± 0.70-**b** | **<0.001 ***** |

*DPI*, days post-prime inoculation; *PBS*, group inoculated with PBS only; *BTV-4*, group inoculated with inactivated BTV-4 only; *AlOOH*, group inoculated with BTV-4 and AlOOH; *ApNPs*, group inoculated with BTV-4 and ApNPs; *MCT*, group inoculated with BTV-4 and MCT; *Md*, median; *IQR*, interquartile range; *O.D.*: optical density; *RMA*: repeated measures ANOVA; *: $p < 0.05$; **: $p < 0.01$; ***: $p < 0.001$; [a] The different letters (*in red*) indicate differences between groups (Duncan's test).

**Table S3.** Effects of inoculations (prime and booster) on group specific antibody levels against BTV.

| Effect of prime inoculation | | | | | | Effect of booster | | | | | |
|---|---|---|---|---|---|---|---|---|---|---|---|
| DPI[a] | PBS *n*= 6 $p_{Dunt}$ | BTV-4 *n*= 6 $p_{Dunt}$ | AlOOH *n*= 6 $p_{Dunt}$ | ApNPs *n*= 6 $p_{Dunt}$ | MCT *n*= 6 $p_{Dunt}$ | DPI[b] | PBS *n*= 6 $p_{Dunt}$ | BTV-4 *n*= 6 $p_{Dunt}$ | AlOOH *n*= 6 $p_{Dunt}$ | ApNPs *n*= 6 $p_{Dunt}$ | MCT *n*= 6 $p_{Dunt}$ |
| ***7-0*** | 0.118 | **<0.001** *** | **<0.001** *** | **<0.001** *** | **<0.001** *** | ***28-21*** | 1.000 | 0.997 | 0.681 | **0.031** * | **0.009** ** |
| ***14-0*** | 0.312 | **<0.001** *** | **<0.001** *** | **<0.001** *** | 0.097 | ***35-21*** | 0.990 | 1.000 | 0.508 | 0.057 | **0.043** * |
| ***21-0*** | 0.108 | **0.004** ** | **0.001** *** | **<0.001** *** | 0.997 | ***42-21*** | 1.000 | 1.000 | 0.782 | 0.130 | 0.068 |
| ***28-0*** | 0.076 | **<0.001** *** | 0.631 | **<0.001** *** | **<0.001** *** | ***49-21*** | 1.000 | 0.988 | 1.000 | 0.084 | 0.060 |
| ***35-0*** | **0.022** * | **<0.001** *** | 0.877 | **<0.001** *** | **<0.001** *** | ***56-21*** | 1.000 | 0.752 | 1.000 | 0.266 | 0.051 |
| ***42-0*** | 0.051 | **<0.001** *** | 0.461 | **<0.001** *** | **<0.001** *** | ***63-21*** | 1.000 | 0.752 | 1.000 | 0.251 | 0.154 |
| ***49-0*** | 0.118 | **<0.001** *** | **0.005** ** | **<0.001** *** | **<0.001** *** | ***70-21*** | 1.000 | 0.076 | 1.000 | 0.074 | **0.030** * |
| ***56-0*** | 0.085 | **<0.001** *** | **0.002** ** | **<0.001** *** | **<0.001** *** | ***77-21*** | 1.000 | **0.032** * | 1.000 | **0.042** * | **0.023** * |
| ***63-0*** | 0.079 | **<0.001** *** | **0.001** *** | **<0.001** *** | **<0.001** *** | ***84-21*** | 1.000 | **0.004** ** | 0.996 | **0.006** ** | **0.010** ** |
| ***70-0*** | **0.043** * | **<0.001** *** | **0.001** *** | **<0.001** *** | **<0.001** *** | ***91-21*** | 0.706 | **0.004** ** | 0.556 | **<0.001** *** | 0.067 |
| ***77-0*** | 0.077 | **<0.001** *** | **<0.001** *** | **<0.001** *** | **<0.001** *** | ***98-21*** | 0.165 | **0.001** *** | 0.378 | **<0.001** *** | **0.022** * |
| ***84-0*** | 0.112 | **<0.001** *** | **<0.001** *** | **<0.001** *** | **<0.001** *** | ***105-21*** | 1.000 | **0.001** *** | 0.231 | **<0.001** *** | **0.028** * |
| ***91-0*** | 0.663 | **<0.001** *** | **<0.001** *** | **<0.001** *** | **<0.001** *** | ***112-21*** | 0.995 | **<0.001** *** | 0.129 | **<0.001** *** | **<0.001** *** |
| ***98-0*** | 0.963 | **<0.001** *** | **<0.001** *** | **<0.001** *** | **<0.001** *** | ***119-21*** | 0.311 | **<0.001** *** | 0.138 | **<0.001** *** | **<0.001** *** |
| ***105-0*** | 0.248 | **<0.001** *** | **<0.001** *** | **<0.001** *** | **<0.001** *** | ***126-21*** | 0.969 | **<0.001** *** | 0.113 | **<0.001** *** | **<0.001** *** |
| ***112-0*** | 0.344 | **<0.001** *** | **<0.001** *** | **<0.001** *** | **<0.001** *** | ***133-21*** | 1.000 | **<0.001** *** | 0.126 | **<0.001** *** | **<0.001** *** |
| ***119-0*** | 0.892 | **<0.001** *** | **<0.001** *** | **<0.001** *** | **<0.001** *** | - | - | - | - | - | - |
| ***126-0*** | 0.427 | **<0.001** *** | **<0.001** *** | **<0.001** *** | **<0.001** *** | - | - | - | - | - | - |
| ***133-0*** | 0.172 | **<0.001** *** | **<0.001** *** | **<0.001** *** | **<0.001** *** | - | - | - | - | - | - |
| ***EB*** | | | 0.07 ± 0.03 | | | | | | | | |

*DPI*, days post-prime inoculation; *Dunt*, Dunnett's test; *: $p < 0.05$; **: $p < 0.01$; ***: $p < 0.001$; [a] All weeks of the experiment are compared with data of all animals at 0 DPI ($n$ = 30) or experimental baseline (*EB*); [b]All weeks of the experiment are compared with 21 DPI within each group. The 21 DPI sample corresponds to serum taken immediately prior to the booster.

**Table S4.** Comparison of neutralizing antibody titers against BTV-4 between groups by date.

| DPI | Group | BTV-4 [$PRNT_{50}$] *Md* ± *IQR*[a] | PBS *n*= 6 | BTV-4 *n*= 6 | AlOOH *n*= 6 $p_{MW}$ | ApNPs *n*= 6 | MCT *n*= 6 |
|---|---|---|---|---|---|---|---|
| 35 | **PBS** | 4.00 ± 4.00-**a** | - | **0.019*** | 0.269 | **0.003**** | **0.003**** |
| | **BTV-4** | 24.00 ± 16.00-**b** | **0.019*** | - | **0.032*** | 0.360 | 0.360 |
| | **AlOOH** | 8.00 ± 4.00-**a** | 0.269 | **0.032** | - | **0.003**** | **0.003**** |
| | **ApNPs** | 48.00 ± 112.00-**b** | **0.003**** | 0.216 | **0.003**** | - | 0.679 |
| | **MCT** | 32.00 ± 48.00-**b** | **0.003**** | 0.360 | **0.003**** | 0.679 | - |
| 70 | **PBS** | 4.00 ± 0.00-**a** | - | **0.002**** | **0.002**** | **0.002**** | **0.002**** |
| | **BTV-4** | 16.00 ± 24.00-**b** | **0.002**** | - | 0.214 | 0.243 | 0.866 |
| | **AlOOH** | 32.00 ± 32.00-**b** | **0.002**** | 0.214 | - | 0.589 | 0.111 |
| | **ApNPs** | 32.00 ± 16.00-**b** | **0.002**** | 0.243 | 0.589 | - | 0.240 |
| | **MCT** | 16.00 ± 16.00-**b** | **0.002**** | 0.866 | 0.111 | 0.679 | - |
| 119 | **PBS** | 1.00 ± 0.00-**a** | - | **0.002**** | **0.002**** | **0.002**** | **0.002**** |
| | **BTV-4** | 48.00 ± 32.00-**b** | **0.002**** | - | 0.553 | 0.553 | 0.082 |
| | **AlOOH** | 32.00 ± 32.00-**b** | **0.002**** | 0.553 | - | 1.000 | 0.132 |
| | **ApNPs** | 32.00 ± 32.00-**b** | **0.002**** | 0.553 | 1.000 | - | 0.180 |
| | **MCT** | 16.00 ± 16.00-**b** | **0.002**** | 0.082 | 0.132 | 0.132 | - |
| 133 | **PBS** | 4.00 ± 0.00-**a** | - | **0.002**** | **0.006**** | **0.002**** | **0.005**** |
| | **BTV-4** | 24.00 ± 24.00-**b** | **0.002**** | - | 0.402 | 0.456 | **0.020*** |
| | **AlOOH** | 64.00 ± 56.00-**bc** | **0.006**** | 0.402 | - | 0.730 | 0.080 |
| | **ApNPs** | 24.00 ± 48.00-**b** | **0.002**** | 0.456 | 0.730 | - | **0.003**** |
| | **MCT** | 8.00 ± 0.00-**c** | **0.005**** | **0.003**** | 0.080 | **0.003**** | - |
| | | | | *Cut-off* = 5 | | | |

*DPI*, days post-prime inoculation; *BTV-4/PRNT50*, highest serum dilution at which the cytopathic effect of bluetongue virus serotype 4 was reduced by 50%; *Md*; median; *IQR*, interquartile range; *MW*, Mann-Whitney test; *, $p < 0.05$; **, $p < 0.01$; ***, $p < 0.001$; [a] The different letters (*in red*) indicate statistical differences between groups.

**Table S5.** Temporal comparison of neutralizing antibody titers against BTV-4 by group.

| Group | 35 | 70 | 119 | 113 | DPI 70-35 | 119-35 | 133-35 | 119-70 | 133-70 | 133-119 | $p_F$ |
|---|---|---|---|---|---|---|---|---|---|---|---|
| | *Md±IQR [BTV-4/PRNT$_{50}$]* | | | | $p_{WSR}$ | | | | | | |
| **PBS**[†] *n= 6* | 4.00 ± 4.00 | 4.00 ± 0.00 | 1.00 ± 0.00 | 4.00 ± 0.00 | 0.157 | 0.023 | 0.157 | 0.014* | 0.317 | 0.027* | 0.001 *** |
| **BTV-4** *n= 6* | 24.00 ± 16.00 | 16.00 ± 24.00 | 48.00 ± 32.00 | 24.00 ± 24.00 | 0.581 | 0.066 | 0.891 | **0.028*** | 0.317 | **0.027*** | **0.014 *** |
| **AlOOH** *n= 6* | 8.00 ± 4.00 | 32.00 ± 32.00 | 32.00 ± 32.00 | 64.00 ± 48.00 | 0.269 | **0.032*** | - | **0.003**** | **0.003**** | 0.461 | **0.016 *** |
| **ApNPs** *n= 6* | 48.00 ± 112.00 | 32.00 ± 16.00 | 32.00 ± 32.00 | 24.00 ± 48.00 | **0.003**** | 0.216 | **0.003**** | - | 0.679 | 0.593 | 0.562 |
| **MCT** *n= 6* | 32.00 ± 48.00 | 16.00 ± 16.00 | 16.00 ± 16.00 | 8.00 ± 0.00 | **0.003**** | 0.360 | **0.003**** | 0.679 | - | **0.046*** | **0.005 **** |
| *Cut-off* = 5 | | | | | | | | | | | |

*DPI*: days post-prime inoculation; *BTV-4/PRNT$_{50}$*: highest serum dilution at which the cytopathic effect of bluetongue virus serotype 4 was reduced by 50%; *Md*, median; *IQR*, interquartile range; *WSR*, Wilcoxon signed-rank test; *F*, Friedman test; [†], All values from the PBS group were below cut off and therefore negative for neutralizing antibodies; *****, $p < 0.05$; ******, $p < 0.01$; *******, $p < 0.001$.

**Table S6.** Red blood cell parameters per group expressed as mean ± standard deviation.

| | G | 0 DPI | 21 DPI | 42 DPI | 63 DPI | 84 DPI | 112 DPI | 133 DPI | R[a] |
|---|---|---|---|---|---|---|---|---|---|
| **RBC** [x$10^6$ µ$L^{-1}$] | **PBS** | 12.98 ± 2.01 | 10.16 ± 2.53 | 10.17 ± 2.62 | 9.77 ± 1.51 | 9.95 ± 2.52 | 11.0 ± 2.92 | 9.93 ± 2.15 | 9.5 - 15.1 |
| | **BTV-4** | 13.25 ± 0.78 | 11.39 ± 0.65 | 11.08 ± 0.37 | 10.75 ± 0.78 | 10.98 ± 0.53 | 12.02 ± 0.87 | 11.01 ± 0.87 | |
| | **AlOOH** | 12.68 ± 1.06 | 10.49 ± 0.71 | 10.51 ± 1.08 | 10.48 ± 1.28 | 9.88 ± 0.78 | 10.58 ± 0.98 | 10.54 ± 1.27 | |
| | **ApNPs** | 13.05 ± 0.53 | 10.47 ± 0.41 | 10.67 ± 1.04 | 10.39 ± 0.54 | 11.23 ± 3.70 | 10.36 ± 0.89 | 9.91 ± 0.96 | |
| | **MCT** | 13.16 ± 1.11 | 10.28 ± 0.63 | 10.65 ± 0.67 | 9.74 ± 0.52 | 10.17 ± 0.87 | 9.77 ± 0.72 | 9.87 ± 0.76 | |
| **Hb** [g d$L^{-1}$] | **PBS** | 12.22 ± 1.92 | 10.25 ± 2.67 | 10.50 ± 2.06 | 9.78 ± 1.57* | 10.32 ± 2.87 | 11.32 ± 3.19 | 10.02 ± 2.36 | 10,00 - 14.90 |
| | **BTV-4** | 12.15 ± 0.69 | 10.88 ± 0.27 | 10.60 ± 0.44 | 10.35 ± 0.89 | 10.58 ± 0.56 | 11.55 ± 0.69 | 10.52 ± 0.83 | |
| | **AlOOH** | 12.38 ± 0.54 | 10.87 ± 0.66 | 10.75 ± 0.70 | 10.62 ± 0.86 | 10.35 ± 0.77 | 11.03 ± 0.63 | 10.60 ± 1.09 | |
| | **ApNPs** | 12.87 ± 1.24 | 10.83 ± 0.79 | 11.05 ± 1.27 | 10.82 ± 0.66 | 11.77 ± 2.90 | 10.90 ± 1.34 | 10.22 ± 8.47 | |
| | **MCT** | 13.00 ± 0.81 | 10.83 ± 0.64 | 11.03 ± 0.77 | 10.15 ± 0.60 | 10.82 ± 0.93 | 10.25 ± 0.56 | 10.05 ± 0.87 | |
| **Hct** [%] | **PBS** | 38.70 ± 7.42 | 30.12 ± 8.56 | 30.25 ± 7.83 | 29.80 ± 5.08 | 31.07 ± 9.92 | 34.47 ± 11.46 | 42.12 ± 2.92 | 27,00 - 42.00 |
| | **BTV-4** | 36.68 ± 3.45 | 30.43 ± 1.84 | 29.12 ± 2.43 | 28.50 ± 3.08 | 29.60 ± 1.92 | 33.03 ± 3.19 | 32.68 ± 1.99 | |
| | **AlOOH** | 39.52 ± 2.96 | 31.93 ± 2.71 | 31.45 ± 3.34 | 31.10 ± 2.52 | 29.53 ± 4.03 | 31.90 ± 3.18 | 33.18 ± 2.18 | |
| | **ApNPs** | 39.18 ± 5.82 | 31.53 ± 3.00 | 31.88 ± 4.21 | 31.32 ± 3.34 | 34.02 ± 8.57 | 31.20 ± 4.8 | 30.93 ± 8.0 | |
| | **MCT** | 42.12 ± 2.92 | 32.68 ± 1.99 | 33.18 ± 2.18 | 30.93 ± 2.13 | 32.32 ± 3.02 | 30.43 ± 2.48 | 30.98 ± 3.44 | |
| **MCV** [fL] | **PBS** | 29.78 ± 4.07 | 29.33 ± 3.98 | 29.58 ± 3.96 | 30.55 ± 3.61 | 30.63 ± 4.84 | 30.62 ± 5.04 | 29.58 ± 5.13 | 24.40 - 32.50 |
| | **BTV-4** | 27.72 ± 2.39 | 26.80 ± 2.48 | 26.30 ± 2.36 | 36.53 ± 2.25 | 27.20 ± 2.32 | 27.57 ± 2.60 | 26.52 ± 2.21 | |
| | **AlOOH** | 31.32 ± 3.08 | 30.50 ± 2.53 | 30.02 ± 2.60 | 29.88 ± 2.79 | 29.90 ± 3.27 | 30.32 ± 3.42 | 29.52 ± 3.22 | |
| | **ApNPs** | 29.70 ± 2.96 | 30.17 ± 2.81 | 29.92 ± 2.93 | 30.17 ± 2.86 | 30.52 ± 2.77 | 30.08 ± 3.26 | 30.07 ± 3.04 | |
| | **MCT** | 32.23 ± 1.20 | 31.88 ± 1.05 | 31.13 ± 0.72 | 31.75 ± 0.81 | 31.77 ± 0.29 | 31.15 ± 0.75 | 31.25 ± 1.51 | |
| **MCH** [pg] | **PBS** | 9.42 ± 0.64 | 10.15 ± 0.76 | 10.43 ± 0.77 | 10.0 ± 0.83 | 10.28 ± 0.79 | 10.20 ± 0.70 | 10.03 ± 0.74 | 8.50 - 11.80 |
| | **BTV-4** | 9.20 ± 0.38 | 9.58 ± 0.54 | 9.58 ± 0.45 | 9.62 ± 0.48 | 9.70 ± 0.55 | 9.62 ± 0.42 | 9.55 ± 0.48 | |
| | **AlOOH** | 9.82 ± 0.53 | 10.38 ± 0.48 | 10.27 ± 0.57 | 10.18 ± 0.52 | 10.50 ± 0.60 | 10.48 ± 0.79 | 10.08 ± 0.62 | |
| | **ApNPs** | 9.77 ± 0.60 | 10.35 ± 0.66 | 10.42 ± 0.57 | 10.55 ± 0.69 | 10.48 ± 0.76 | 10.38 ± 0.74 | 10.13 ± 0.26 | |
| | **MCT** | 9.97 ± 0.36 | 10.57 ± 0.24 | 10.35 ± 0.30 | 10.45 ± 0.19 | 10.63 ± 0.36 | 10.52 ± 0.25 | 10.13 ± 0.26 | |
| **MCHC** [%] | **PBS** | 31.90 ± 2.73↓ | 34.87 ± 2.56 | 35.72 ± 5.22 | 32.90 ± 1.26 | 33.98 ± 3.24 | 33.88 ± 4.03 | 34.53 ± 4.33 | 32.30 - 42.00 |
| | **BTV-4** | 33.25 ± 1.92 | 35.83 ± 2.15 | 36.53 ± 1.87 | 36.42 ± 1.42 | 35.80 ± 1.29 | 35.10 ± 2.03 | 36.13 ± 1.77 | |
| | **AlOOH** | 31.43 ± 1.76↓ | 34.12 ± 1.51 | 34.33 ± 1.91 | 34.17 ± 1.79 | 35.37 ± 3.22 | 34.70 ± 1.83 | 34.33 ± 1.90 | |
| | **ApNPs** | 33.07 ± 1.80 | 34.72 ± 1.56 | 34.72 ± 1.37 | 34.70 ± 1.79 | 34.65 ± 1.14 | 35.08 ± 1.48 | 34.60 ± 1.65 | |
| | **MCT** | 30.88 ± 0.59↓ | 33.15 ± 1.20 | 33.23 ± 0.63 | 32.85 ± 0.83 | 33.50 ± 1.13 | 33.77 ± 1.24 | 32.53 ± 1.29 | |

*DPI*, days post-prime inoculation, *RBC*, red blood cell count; *Hb*, hemoglobin; *Hct*, hematocrit; *MCV*, mean corpuscular volume; *MCH*, mean corpuscular hemoglobin; *MCHC*, mean corpuscular hemoglobin concentration; *R*, general reference range; ↓: mean value below the reference range; [a]Reference range from the IDEXX Procyte Dx hematological analyzer for sheep.

**Table S7.** White blood cell and platelet counts per group expressed as mean ± standard deviation.

| | Grupo | 0 DPI | 21 DPI | 42 DPI | 63 DPI | 84 DPI | 112 DPI | 133 DPI | R[a] |
|---|---|---|---|---|---|---|---|---|---|
| **WBC** [x10$^3$ μL$^{-1}$] | **PBS** | 7.88 ± 0.97 | 6.94 ± 0.67 | 6.69 ± 1.21 | 7.23 ± 0.76 | 7.64 ± 0.79 | 8.00 ± 1.40 | 7.82 ± 1.74 | 5.1-14.1 |
| | **BTV-4** | 8.39 ± 0.82 | 8.04 ± 1.11 | 8.22 ± 0.71 | 7.68 ± 0.55 | 7.73 ± 0.37 | 8.40 ± 0.61 | 8.75 ± 0.98 | |
| | **AlOOH** | 7.77 ± 0.95 | 7.86 ± 1.19 | 7.61 ± 1.30 | 7.27 ± 0.91 | 7.64 ± 1.36 | 8.15 ± 0.89 | 7.75 ± 1.27 | |
| | **ApNPs** | 6.81 ± 0.73 | 7.23 ± 1.62 | 6.92 ± 1.31 | 7.47 ± 1.64 | 7.34 ± 1.66 | 7.15 ± 1.16 | 7.16 ± 1.03 | |
| | **MCT** | 8.90 ± 1.80 | 8.00 ± 1.20 | 8.50 ± 1.50 | 8.80 ± 1.80 | 8.80 ± 1.80 | 9.30 ± 1.20 | 8.91 ± 0.96 | |
| **Lym** [x10$^3$ μL$^{-1}$] | **PBS** | 4.74 ± 0.83 | 4.27 ± 0.70 | 4.25 ± 0.96 | 4.37 ± 0.69 | 4.35 ± 0.65 | 4.43 ± 0.93 | 3.93 ± 1.04 | 2.5-9.6 |
| | **BTV-4** | 5.19 ± 0.53 | 4.39 ± 0.68 | 5.27 ± 0.48 | 4.64 ± 0.62 | 4.42 ± 0.32 | 4.35 ± 0.45 | 4.47 ± 0.56 | |
| | **AlOOH** | 4.29 ± 1.69 | 4.07 ± 0.82 | 4.33 ± 0.61 | 4.13 ± 0.60 | 3.95 ± 0.66 | 4.64 ± 0.71 | 4.49 ± 0.52 | |
| | **ApNPs** | 3.77 ± 0.60 | 4.04 ± 0.95 | 4.35 ± 0.86 | 4.41 ± 0.92 | 3.77 ± 1.48 | 4.05 ± 1.13 | 4.06 ± 0.76 | |
| | **MCT** | 5.23 ± 1.68 | 5.86 ± 3.28 | 5.65 ± 1.31 | 5.38 ± 0.98 | 4.93 ± 1.34 | 4.78 ± 0.60 | 4.57 ± 1.07 | |
| **Neu** [x10$^3$ μL$^{-1}$] | **PBS** | 2.11 ± 0.46 | 1.98 ± 0.47 | 1.74 ± 0.45* | 2.01 ± 0.48 | 2.43 ± 1.18 | 2.88 ± 0.87 | 2.43 ± 1.18 | 1.17-6.1 |
| | **BTV-4** | 2.05 ± 0.41 | 2.60 ± 0.54 | 2.06 ± 0.20 | 2.08 ± 0.22 | 2.11 ± 0.32 | 2.76 ± 0.41 | 3.30 ± 0.68 | |
| | **AlOOH** | 2.45 ± 1.44 | 2.45 ± 0.74 | 2.32 ± 0.60 | 2.25 ± 0.51 | 2.43 ± 0.88 | 2.54 ± 0.66 | 2.27 ± 0.62 | |
| | **ApNPs** | 2.11 ± 0.53 | 2.19 ± 0.56 | 1.81 ± 0.38 | 2.43 ± 0.82 | 2.69 ± 0.71 | 2.48 ± 0.40 | 2.32 ± 0.47 | |
| | **MCT** | 2.51 ± 1.16 | 2.31 ± 0.92 | 2.06 ± 0.83 | 3.17 ± 1.67 | 2.80 ± 0.99 | 3.55 ± 1.15 | 3.12 ± 0.70 | |
| **Eos** [x10$^3$ μL$^{-1}$] | **PBS** | 0.26 ± 0.28 | 0.14 ± 0.11 | 0.20 ± 0.08 | 0.12 ± 0.07 | 0.22 ± 0.16 | 0.14 ± 0.08 | 0.18 ± 0.11 | 0.1-1.0 |
| | **BTV-4** | 0.12 ± 0.09 | 0.16 ± 0.10 | 0.13 ± 0.08 | 0.17 ± 0.09 | 0.21 ± 0.11 | 0.17 ± 0.08 | 0.15 ± 0.06 | |
| | **AlOOH** | 0.10 ± 0.08 | 0.17 ± 0.07 | 0.14 ± 0.08 | 0.10 ± 0.03 | 0.12 ± 0.10 | 0.15 ± 0.05 | 0.17 ± 0.05 | |
| | **ApNPs** | 0.07 ± 0.03 | 0.13 ± 0.04 | 0.10 ± 0.03 | 0.10 ± 0.05 | 0.11 ± 0.05 | 0.08 ± 0.04 | 0.11 ± 0.03 | |
| | **MCT** | 0.12 ± 0.10 | 0.13 ± 0.09 | 0.14 ± 0.08 | 0.16 ± 0.07 | 0.13 ± 0.06 | 0.23 ± 0.10 | 0.17 ± 0.07 | |
| **Bas** [x10$^3$ μL$^{-1}$] | **PBS** | 0.05 ± 0.04 | 0.06 ± 0.04 | 0.04 ± 0.02 | 0.05 ± 0.02 | 0.07 ± 0.03 | 0.08 ± 0.06 | 0.05 ± 0.03 | 0.0-0.1 |
| | **BTV-4** | 0.07 ± 0.03 | 0.11 ± 0.04 | 0.04 ± 0.02 | 0.04 ± 0.02 | 0.10 ± 0.02 | 0.08 ± 0.04 | 0.06 ± 0.02 | |
| | **AlOOH** | 0.04 ± 0.04 | 0.05 ± 0.03 | 0.05 ± 0.01 | 0.06 ± 0.02 | 0.07 ± 0.02 | 0.05 ± 0.03 | 0.04 ± 0.03 | |
| | **ApNPs** | 0.04 ± 0.03 | 0.07 ± 0.04 | 0.03 ± 0.02 | 0.04 ± 0.01 | 0.07 ± 0.03 | 0.05 ± 0.03 | 0.04 ± 0.03 | |
| | **MCT** | 0.03 ± 0.03 | 0.07 ± 0.05 | 0.05 ± 0.03 | 0.06 ± 0.03 | 0.07 ± 0.06 | 0.06 ± 0.05 | 0.09 ± 0.04 | |
| **PLT** [x10$^3$ μL$^{-1}$] | **PBS** | 352 ± 67 | 267 ± 124↓ | 297 ± 66↓ | 313 ± 59 | 368 ± 108 | 266 ± 192↓ | 297 ± 110↓ | 301-922 |
| | **BTV-4** | 360 ± 65 | 248 ± 61↓ | 353 ± 52 | 331 ± 58 | 342 ± 47 | 275 ± 91↓ | 330 ± 64 | |
| | **AlOOH** | 455 ± 80 | 331 ± 110 | 376 ± 80 | 414 ± 64 | 402 ± 197 | 312 ± 43 | 350 ± 47 | |
| | **ApNPs** | 480 ± 130 | 244 ± 105↓ | 354 ± 127 | 327 ± 71 | 310 ± 112 | 257 ± 73↓ | 309 ± 130 | |
| | **MCT** | 424 ± 59 | 316 ± 199 | 397 ± 49 | 385 ± 45 | 361 ± 208 | 345 ± 126 | 391 ± 60 | |

*DPI*, days post-prime inoculation, *WBC*, white blood cell count; *Lym*, lymphocytes; *Neu*, neutrophils; *Eos*, eosinophils; *Bas*, basophils; *PLT*, platelets; *R*, general reference range; ↓, mean value below the reference range. [a]Reference range from the IDEXX Procyte Dx hematological analyzer for sheep.

**Table S8.** Biochemical parameters analyzed per group expressed as mean ± standard deviation.

| | Group | 0 DPI | 7 DPI | 28 DPI | 133 DPI | R[a] |
|---|---|---|---|---|---|---|
| **$Ca^{2+}$** [mg $dL^{-1}$] | **PBS** | 9.32 ± 0.24 | 9.33 ± 0.12 | 9.40 ± 0.21 | 9.57 ± 0.23 | 9.1-10.8 |
| | **BTV-4** | 9.57 ± 0.21 | 9.68 ± 0.23 | 9.52 ± 0.21 | 9.73 ± 0.10 | |
| | **AlOOH** | 9.33 ± 0.10 | 9.38 ± 0.12 | 9.37 ± 0.10 | 9.28 ± 0.22 | |
| | **ApNPs** | 9.57 ± 1.00 | 9.43 ± 0.70 | 9.30 ± 0.58 | 9.45 ± 0.34 | |
| | **MCT** | 9.60 ± 0.68 | 9.60 ± 0.68 | 9.33 ± 0.68 | 9.35 ± 0.80 | |
| **Phos** [mg $dL^{-1}$] | **PBS** | 5.08 ± 0.72 | 5.28 ± 0.66 | 5.02 ± 0.52 | 5.13 ± 0.55 | 4.0-8.9 |
| | **BTV-4** | 5.80 ± 0.75 | 5.30 ± 0.77 | 5.42 ± 0.74 | 5.62 ± 0.77 | |
| | **AlOOH** | 5.80 ± 0.75 | 5.93 ± 0.72 | 5.82 ± 0.71 | 5.77 ± 0.56 | |
| | **ApNPs** | 5.85 ± 1.00 | 5.75 ± 0.70 | 5.73 ± 0.58 | 5.83 ± 0.34 | |
| | **MCT** | 5.68 ± 0.68 | 5.38 ± 0.41 | 5.83 ± 0.80 | 5.83 ± 0.80 | |
| **Crea** [mg $dL^{-1}$] | **PBS** | 0.88 ± 0.09 | 0.76 ± 0.18 | 0.96 ± 0.26 | 0.98 ± 0.15 | 0.6-1.5 |
| | **BTV-4** | 0.90 ± 0.19 | 0.82 ± 0.12 | 1.28 ± 0.18 | 0.97 ± 0.15 | |
| | **AlOOH** | 0.97 ± 0.12 | 0.92 ± 0.12 | 0.92 ± 0.12 | 0.93 ± 0.14 | |
| | **ApNPs** | 0.85 ± 0.08 | 0.90 ± 0.14 | 0.90 ± 0.14 | 0.82 ± 0.13 | |
| | **MCT** | 0.92 ± 0.08 | 0.83 ± 0.10 | 0.83 ± 0.10 | 0.95 ± 0.10 | |
| **BUN** [mg $dL^{-1}$] | **PBS** | 3.50 ± 1.38↓ | 5.50 ± 2.26 | 11.00 ± 3.58 | 4.00 ± 1.79↓ | 5.0-20.0 |
| | **BTV-4** | 5.50 ± 2.26 | 5.83 ± 2.04 | 14.33 ± 1.86 | 4.00 ± 1.10↓ | |
| | **AlOOH** | 5.67 ± 1.63 | 4.17 ± 1.17↓ | 5.67 ± 1.03 | 3.17 ± 1.17↓ | |
| | **ApNPs** | 3.83 ± 1.17↓ | 5.67 ± 1.03 | 8.00 ± 2.97 | 10.50 ± 1.87 | |
| | **MCT** | 3.83 ± 1.47↓ | 3.17 ± 1.17↓ | 9.33 ± 4.13 | 8.50 ± 1.76 | |
| **GGT** [U $L^{-1}$] | **PBS** | 79.17 ± 20.93 | 69.17 ± 14.40 | 77.50 ± 7.87 | 90.83 ± 18.64 | 33.0-55.0 |
| | **BTV-4** | 71.17 ± 10.68 | 68.50 ± 11.52 | 90.83 ± 13.85 | 70.33 ± 18.15 | |
| | **AlOOH** | 77.00 ± 16.78 | 74.83 ± 9.93 | 73.67 ± 12.88 | 80.67 ± 15.83 | |
| | **ApNPs** | 63.00 ± 9.47 | 64.83 ± 13.06 | 70.83 ± 17.36 | 74.00 ± 9.55 | |
| | **MCT** | 74.83 ± 8.98 | 65.33 ± 10.46 | 79.50 ± 12.14 | 87.00 ± 19.80 | |
| **ALT** [U $L^{-1}$] | **PBS** | 31.33 ± 4.63↑ | 38.33 ± 4.72↑ | 35.00 ± 7.56↑ | 31.17 ± 2.79↑ | 5.0-17.0 |
| | **BTV-4** | 29.83 ± 4.71↑ | 38.50 ± 7.23↑ | 44.83 ± 6.40↑ | 33.67 ± 9.52↑ | |
| | **AlOOH** | 37.67 ± 5.99↑ | 36.17 ± 6.31↑ | 38.00 ± 10.77↑ | 38.00 ± 10.77↑ | |
| | **ApNPs** | 23.00 ± 6.07↑ | 35.67 ± 6.65↑ | 25.00 ± 2.61↑ | 25.00 ± 2.61↑ | |
| | **MCT** | 32.50 ± 4.97↑ | 34.50 ± 4.64↑ | 32.50 ± 11.95↑ | 32.5 ± 11.95↑ | |
| **AST** [U $L^{-1}$] | **PBS** | 112.5 ± 16.02↑ | 102.50 ± 14.02↑ | 101.83 ± 27.19↑ | 107.50 ± 10.80↑ | 40.0-96.0 |
| | **BTV-4** | 98.17 ± 4.71↑ | 107.50 ± 13.52↑ | 145.33 ± 24.61↑ | 118.33 ± 23.23↑ | |
| | **AlOOH** | 115.33 ± 16.86↑ | 145.33 ± 24.61↑ | 109.50 ± 7.40↑ | 123.33 ± 41.58↑ | |
| | **ApNPs** | 109.67 ± 11.72↑ | 139.00 ± 37.29↑ | 139.00 ± 37.29↑ | 105.83 ± 11.75↑ | |
| | **MCT** | 103.50 ± 17.49↑ | 109.50 ± 20.74↑ | 109.50 ± 20.74↑ | 131.33 ± 43.50↑ | |

*Table S8 to be continued on the next page*

**Table S8.** Biochemical parameters analyzed per group expressed as mean ± standard deviation.

| | Group | 0 DPI | 7 DPI | 28 DPI | 133 DPI | R[a] |
|---|---|---|---|---|---|---|
| **Tbil** [a)] [mg $dL^{-1}$] | **PBS** | 0.13 ± 0.05 | 0.12 ± 0.04 | 0.13 ± 0.05 | 0.17 ± 0.10 | 0.1-0.4 |
| | **BTV-4** | 0.15 ± 0.08 | 0.12 ± 0.04 | 0.12 ± 0.04 | 0.15 ± 0.08 | |
| | **AlOOH** | 0.32 ± 0.12 | 0.28 ± 0.17 | 0.25 ± 0.10 | 0.30 ± 0.17 | |
| | **ApNPs** | 0.12 ± 0.04 | 0.13 ± 0.08 | 0.12 ± 0.04 | 0.15 ± 0.12 | |
| | **MCT** | 0.22 ± 0.04 | 0.22 ± 0.10 | 0.27 ± 0.08 | 0.45 ± 0.22↑ | |

*DPI*, days post-prime inoculation, *$Ca^{2+}$*, total calcium, *Phos*, phosphorus; *Crea*, creatinine; *BUN*, blood urea nitrogen; *GGT*, gamma-glutamyl transferase; *Tbil*, total bilirubin; *ALT*, alanine aminotransferase; *AST*; aspartate aminotransferase; *R*, general reference range; ↓ or ↑, mean value below or above, respectively, the reference range. [a]Reference range from the IDEXX Catalyst biochemical analyzer for sheep.

**Table S9.** Data from exploratory clinical analyses per group expressed as mean ± standard deviation

| Group | RR (rpm) | | HR (bpm) | | T (ºC) | |
|---|---|---|---|---|---|---|
| **0 DPI** | | | | | | |
| | Mean ± SD | **Ref** [a] | Mean ± SD | **Ref** [a] | Mean ± SD | **Ref** [a] |
| **PBS** | 48.0 ± 13.4↑ | | 102.7 ± 25.8 | | 39.5 ± 0.5 | |
| **BTV-4** | 50.7 ± 10.01↑ | | 108.0 ± 17.2 | | 39.9 ± 0.4 | |
| **AlOOH** | 71.3 ± 20.1↑ | 16.00-40.00 | 118.7 ± 17.5 | 70.00-290.00 | 40.0 ± 0.18* | 38.30-39.90 |
| **ApNPs** | 78 ± 27.6↑ | | 124 ± 29.8 | | 39.9 ± 0.2 | |
| **MCT** | 77.3 ± 24.2↑ | | 124.0 ± 29.8 | | 39.89 ± 0.16 | |
| **28 DPI** | | | | | | |
| **Group** | **RR** (rpm) | | **HR** (bpm) | | **T** (ºC) | |
| | Mean ± SD | **Ref** [a] | Mean ± SD | **Ref** [a] | Mean ± SD | **Ref** [a] |
| **PBS** | 38.0 ± 7.5 | | 88.7 ± 15.5 | | 39.5 ± 0.5 | |
| **BTV-4** | 46.7 ± 5.5↑ | | 95.0 ± 29.6 | | 39.6 ± 0.3 | |
| **AlOOH** | 33.33 ± 4.13 | 16.00-40.00 | 100.0 ± 15.6 | 70.00-290.00 | 39.15 ± 0,24 | 38.30-39.90 |
| **ApNPs** | 42.07 ± 12.07↑ | | 92.0 ± 12.65 | | 39.50 ± 0.21 | |
| **MCT** | 34.7 ± 7.4 | | 100.7 ± 25 | | 39.34 ± 0.25 | |

*DPI*, days post-prime inoculation; *RR*, respiratory rate; *HR*, heart rate; *T*; temperature; *SD*, standard deviation; *Ref*; general physiological reference range; ↑, mean value above the reference range [a]General reference ranges were extracted from Reece et al. (2015).

**Table S10.** Prime inoculation. Relative frequencies and statistical analysis by grade, group, and date of injection sites (ISs).

| DPI | Group | Grade [% ][a] 0 | 1 | 2 | 3 | $p_{LR}$ |
|---|---|---|---|---|---|---|
| 7 | AlOOH | 16.7 | 0 | 0 | *83.3↑↑↑* | 0.001 ** |
| | ApNPs | 66.7 | 33.3 | 0 | 0 | |
| | MCT | 33.3 | 16.7 | *50↑↑* | 0 | |
| 9 | AlOOH | 0 | *0↓* | 0 | *100↑↑↑* | <0.001 *** |
| | ApNPs | 0 | *83.3↑↑* | 16.7 | *0↓* | |
| | MCT | 16.7 | 33.3 | *50↑* | *0↓* | |
| 12 | AlOOH | *0↓↓* | - | 0 | *100↑↑↑* | <0.001 *** |
| | ApNPs | *100↑↑* | - | 0 | *0↓* | |
| | MCT | 50.0 | - | *50↑↑* | *0↓* | |
| 13 | AlOOH | *0↓↓* | 16.7 | 0 | *83.3↑↑↑* | 0.001 ** |
| | ApNPs | 66.7 | 33.3 | 0 | 0 | |
| | MCT | *83.3↑* | 0 | 16.7 | 0 | |
| 15 | AlOOH | *0↓↓↓* | - | - | *100↑↑↑* | <0.001 *** |
| | ApNPs | *100↑* | - | - | *100↓* | |
| | MCT | *100↑* | - | - | *100↓* | |
| 18 | AlOOH | *16.7↓↓↓* | 16.7 | *50.0↓↓↓* | 16.7 | 0.016 * |
| | ApNPs | 16.7 | 16.7 | 50.0 | 16.7 | |
| | MCT | *100↑↑* | 0 | 0 | 0 | |
| 22 | AlOOH | *0↓↓↓* | - | *83.3↑↑↑* | 16.7 | <0.001 *** |
| | ApNPs | *100↑* | - | 0 | 0 | |
| | MCT | *100↑* | - | 0 | 0 | |
| 25 | AlOOH | *0↓↓↓* | 16.7 | *33.3↑* | *50.0↑↑* | 0.001 *** |
| | ApNPs | *100↑* | 0 | 0 | 0 | |
| | MCT | *100↑* | 0 | 0 | 0 | |
| 27 | AlOOH | 0 | 66.7 | 16.7 | 16.7 | >0.05 |
| | ApNPs | 33.3 | 16.7 | 50.0 | 0 | |
| | MCT | 16.7 | 16.7 | 66.7 | 0 | |
| 31 | AlOOH | *0↓↓↓* | *83.3↑↑↑* | 16.7 | - | <0.001 *** |
| | ApNPs | *100↑* | 0 | 0 | - | |
| | MCT | *100↑* | 0 | 0 | - | |
| 35 | AlOOH | *0↓↓↓* | *66.7↑↑* | *33.3↑* | - | <0.001 *** |
| | ApNPs | *100↑* | 0 | 0 | - | |
| | MCT | *100↑* | 0 | 0 | - | |
| 38 | AlOOH | *16.7↓↓↓* | *50.0↑↑* | *33.3↑* | - | 0.003 ** |
| | ApNPs | 100 | 0 | 0 | - | |
| | MCT | 100 | 0 | 0 | - | |

| DPI | Group | Grade [% ][a] 0 | 1 | 2 | 3 | $p_{LR}$ |
|---|---|---|---|---|---|---|
| 45 | AlOOH | *0↓↓↓* | *83.3↑↑↑* | 16.7 | - | <0.001 *** |
| | ApNPs | *100↑* | 0 | 0 | - | |
| | MCT | *100↑* | 0 | 0 | - | |
| 49 | AlOOH | *16.7↓↓↓* | *66.7↑↑* | 16.7 | - | <0.001 *** |
| | ApNPs | 100 | 0 | 0 | - | |
| | MCT | 100 | 0 | 0 | - | |
| 63 | AlOOH | *33.3↓↓* | *66.7↑↑* | - | - | 0.003 ** |
| | ApNPs | 100 | 100 | - | - | |
| | MCT | 100 | 0 | - | - | |
| 70 | AlOOH | *16.7↓↓↓* | *83.3↑↑↑* | - | - | <0.001 *** |
| | ApNPs | 100 | 0 | - | - | |
| | MCT | 100 | 0 | - | - | |
| 75 | AlOOH | *16.7↓↓↓* | *83.3↑↑↑* | - | - | <0.001 *** |
| | ApNPs | 100 | 0 | - | - | |
| | MCT | 100 | 0 | - | - | |
| 83 | AlOOH | *16.7↓↓↓* | *83.3↑↑↑* | - | - | <0.001 *** |
| | ApNPs | 100 | 0 | - | - | |
| | MCT | 100 | 0 | - | - | |
| 98 | AlOOH | *33.3↓↓* | *66.7↑↑* | - | - | 0.003 ** |
| | ApNPs | 100 | 0 | - | - | |
| | MCT | 100 | 0 | - | - | |
| 105 | AlOOH | *50.0↓↓* | *50.0↑↑* | - | - | 0.019 * |
| | ApNPs | 100 | 0 | - | - | |
| | MCT | 100 | 0 | - | - | |
| 117 | AlOOH | *50.0↓↓* | *50.0↑↑* | - | - | 0.019 * |
| | ApNPs | 100 | 0 | - | - | |
| | MCT | 100 | 0 | - | - | |
| 126 | AlOOH | 83.3 | 16.7 | - | - | >0.05 |
| | ApNPs | 100 | 0 | - | - | |
| | MCT | 100 | 0 | - | - | |
| 133 | AlOOH | 18.8 ↓↓↓ | 43.5 ↑↑↑ | 13.0 ↑↑ | 24.6 ↑↑↑ | <0.001 *** |
| | ApNPs | 88.4 ↑↑↑ | 8.7 ↓↓↓ | 2.9 ↓↓↓ | 0 ↓↓↓ | |
| | MCT | 87.0 ↑↑↑ | 2.9 ↓↓↓ | 10.1 | 0 ↓↓↓ | |

*DPI;* days post-prime inoculation*; LR*, Likelihood-Ratio; [a]Data in red italics indicate significance. Arrows indicate the direction and magnitude of significance based on analysis of adjusted residuals: one arrow (*$p$ < 0.05), two arrows (**$p$ < 0.01), three arrows (***$p$ < 0.001).

**Table S11.** Booster inoculation. Relative frequencies and statistical analysis by grade, group, and date of injection sites (ISs).

| DPI | Group | Grade [%][a] 0 | 1 | 2 | 3 | $p_{LR}$ | DPI | Group | Grade [%][a] 0 | 1 | 2 | 3 | $p_{LR}$ |
|---|---|---|---|---|---|---|---|---|---|---|---|---|---|
| | AlOOH | 100 | 0 | 0 | 0 | | | AlOOH | ***50↓↓*** | ***50↑↑*** | - | - | |
| **23** | ApNPs | 66.7 | 16.7 | 0 | 16.7 | >0.05 | **63** | ApNPs | 100 | 0 | - | - | **0.019 *** |
| | MCT | 33.3 | 50.0 | 16.7 | 0 | | | MCT | 100 | 0 | - | - | |
| | AlOOH | 66.7 | 16.7 | 16.7 | 0 | | | AlOOH | ***50↓↓*** | ***50↑↑*** | - | - | |
| **24** | ApNPs | 50.0 | 16.7 | 0 | 33.3 | >0.05 | **70** | ApNPs | 100 | 0 | - | - | **0.019 *** |
| | MCT | 16.7 | 16.7 | 0 | 66.7 | | | MCT | 100 | 0 | - | - | |
| | AlOOH | ***83.3↑↑*** | 16.7 | - | ***0↓*** | | | AlOOH | ***33.3↓↓*** | ***66.7↑↑*** | - | - | |
| **25** | ApNPs | 16.7 | 33.3 | - | 50.0 | **0.032 *** | **75** | ApNPs | 100 | 0 | - | - | **0.019 *** |
| | MCT | 16.7 | 16.7 | - | 66.7 | | | MCT | 100 | 0 | - | - | |
| | AlOOH | 16.7 | ***83.3↑↑*** | ***0↓*** | - | | | AlOOH | ***50↓↓*** | ***50↑↑*** | - | - | |
| **27** | ApNPs | 33.3 | 16.7 | 50.0 | - | **0.032 *** | **83** | ApNPs | 100 | 0 | - | - | **0.019 *** |
| | MCT | 16.7 | 16.7 | 66.7 | - | | | MCT | 100 | 0 | - | - | |
| | AlOOH | 16.7 | 16.7 | 66.7 | 0 | | | AlOOH | 83.3 | 16.7 | - | - | |
| **31** | ApNPs | 16.7 | 16.7 | 16.7 | 50.0 | >0.05 | **98** | ApNPs | 100 | 0 | - | - | >0.05 |
| | MCT | 0 | 50.0 | 16.7 | 33.3 | | | MCT | 100 | 0 | - | - | |
| | AlOOH | 16.7 | 83.3 | 0 | 0 | | | AlOOH | 66.7 | 33.3 | - | - | |
| **35** | ApNPs | 16.7 | 33.3 | 33.3 | 16.7 | >0.05 | **105** | ApNPs | 100 | 0 | - | - | >0.05 |
| | MCT | 33.3 | 16.7 | 33.3 | 16.7 | | | MCT | 100 | 0 | - | - | |
| | AlOOH | 66.7 | 33.3 | 0 | 0 | | | AlOOH | 83.3 | 16.7 | - | - | |
| **38** | ApNPs | 33.3 | 50.0 | 16.7 | 0 | >0.05 | **117** | ApNPs | 100 | 0 | - | - | >0.05 |
| | MCT | 83.3 | 0 | 0 | 16.7 | | | MCT | 100 | 0 | - | - | |
| | AlOOH | 0.0 | 33.3 | 0 | - | | | AlOOH | 83.3 | 16.7 | - | - | |
| **45** | ApNPs | 33.3 | 50.0 | 16.7 | - | >0.05 | **126** | ApNPs | 100 | 0 | - | - | >0.05 |
| | MCT | 100 | 0 | 0 | - | | | MCT | 100 | 0 | - | - | |
| | AlOOH | ***16.7↓↓*** | ***83.3↑↑*** | - | - | | | AlOOH | 83.3 | 16.7 | - | - | |
| **49** | ApNPs | 83.3 | 16.7 | - | - | **0.002 **** | **133** | ApNPs | 100 | 0 | - | - | >0.05 |
| | MCT | ***100↑*** | ***0↓*** | - | - | | | MCT | 100 | 0 | - | - | |

*DPI;* days post-prime inoculation*; LR,* Likelihood-Ratio test; [a] Data in red italics indicate significance. Arrows indicate the direction and magnitude of significance based on analysis of adjusted residuals: one arrow (*$p < 0.05$), two arrows (**$p < 0.01$), three arrows (***$p < 0.001$).

**Table S12.** Regional lymph nodes. Comparative analysis of cortex-paracortex thickening (CPT), secondary follicles (SF), medullary plasmacytosis (MP), medullary histiocytosis (MH), and follicular hyalinosis (FH) by group.

| Histol cat. | Side | Group | **Grade** (%, absolute frequencies)[a] 0 | 1 | 2 | 3 | $p_{LR/\chi^2}$ |
|---|---|---|---|---|---|---|---|
| CPT | Right (prime) | PBS | 0 (0/12) | **41.7 (5/12)↑** | 41.7 (5/12) | **16.7 (2/12)↓** | **0.001**** (LR) |
| | | BTV-4 | 0 (0/11) | 0 (0/11) | 54.5 (6/11) | 45.5 (5/11) | |
| | | AlOOH | 0 (0/11) | 0 (0/11) | 18.2 (2/11) | **81.8 (9/11)↑↑** | |
| | | ApNPs | 0 (0/12) | 33.3 (4/12) | 50.0 (6/12) | **16.7 (2/12)↓** | |
| | | MCT | 0 (0/11) | 9.1 (1/11) | 18.2 (2/11) | **72.7 (8/11)↑** | |
| | Left (booster) | PBS | 0 (0/12) | 25.0 (3/12) | 50.0 (6/12) | 25.0 (3/12) | **0.006**** (LR) |
| | | BTV-4 | 0 (0/12) | 0 (0/12) | 41.7 (5/12) | 58.3 (7/12) | |
| | | AlOOH | 0 (0/12) | 0 (0/12) | 33.3 (4/12) | 66.7 (8/12) | |
| | | ApNPs | 0 (0/12) | **33.3 (4/12)↑** | 50.0 (6/12) | **16.7 (2/12)↓** | |
| | | MCT | 0 (0/11) | 18.2 (2/11) | **9.1 (1/11)↓** | 72.7 (8/11) | |
| | **Total** | PBS | 0 (0/24) | 33.3 (8/24) | 45.8 (11/24) | **20.8 (5/24)↓↓** | **<0.001***** (LR) |
| | | BTV-4 | 0 (0/23) | 0 (0/23) | 47.8 (11/23) | 52.2 (12/23) | |
| | | AlOOH | 0 (0/23) | 0 (0/23) | 26.1 (6/23) | **73.9 (17/23)↑↑** | |
| | | ApNPs | 0 (0/24) | 33.3 (8/24) | 50.0 (12/24) | **16.7 (4/24)↓↓↓** | |
| | | MCT | 0 (0/22) | 13.6 (3/22) | 13.6 (3/22) | **72.7 (8/11)↑↑** | |
| SF | Right (prime) | PBS | 8.3 (1/12) | 58.3 (7/12) | 33.3 (4/12) | 0 (0/12) | **0.011*** (LR) |
| | | BTV-4 | 18.2 (2/11) | 18.2 (2/11) | 54.5 (6/11) | 9.1 (1/11) | |
| | | AlOOH | 0 (0/11) | **9.1 (1/11)↓** | 45.5 (5/11) | **45.5 (5/11)↑** | |
| | | ApNPs | 16.7 (2/12) | 58.3 (7/12) | 16.7 (2/12) | 8.3 (1/12) | |
| | | MCT | 0 (0/11) | 27.3 (3/11) | 36.4 (4/11) | 36.4 (4/11) | |
| | Left (booster) | PBS | 8.3 (1/12) | 25.0 (3/12) | 58.3 (7/12) | 8.3 (1/12) | 0.172 (LR) |
| | | BTV-4 | 0 (0/12) | 66.7 (8/12) | 25.0 (3/12) | 8.3 (1/12) | |
| | | AlOOH | 0 (0/12) | 33.3 (4/12) | 50.0 (6/12) | 16.7 (2/12) | |
| | | ApNPs | 8.3 (1/12) | 66.7 (8/12) | 25.0 (3/12) | 0 (0/12) | |
| | | MCT | 0 (0/11) | 54.5 (6/11) | 18.2 (2/11) | 27.3 (3/11) | |
| | **Total** | PBS | 8.3 (2/24) | 41.7 (10/24) | 45.8 (11/24) | 4.2 (1/24) | **0.006**** (LR) |
| | | BTV-4 | 8.7 (2/23) | 43.5 (10/23) | 39.1 (9/23) | 8.7 (2/23) | |
| | | AlOOH | 0 (0/23) | **21.7 (5/23)↓** | 47.8 (11/23) | **30.4 (7/23)↑** | |
| | | ApNPs | 12.5 (3/24) | **62.5 (15/24)↑** | 20.8 (5/24) | 4.2 (1/24) | |
| | | MCT | 0 (0/22) | 40.9 (9/22) | 27.3 (6/23) | **31.8 (7/22)↑** | |
| MP | Right (prime) | PBS | 8.3 (1/12) | 66.7 (8/12) | 25.0 (3/12) | 0 (0/12) | **0.033*** |
| | | BTV-4 | 9.1 (1/11) | 63.6 (7/11) | 18.2 (2/11) | 9.1 (1/11) | |
| | | AlOOH | 0 (0/11) | 54.5 (6/11) | 27.3 (3/11) | 18.2 (2/11) | |
| | | ApNPs | 16.7 (2/12) | **83.3 (10/12)↑** | **0 (0/12)↓** | 0 (0/12) | |
| | | MCT | 27.3 (3/11) | **18.2 (2/11)↓↓** | 36.4 (4/11) | 18.2 (2/11) | |
| | Left (booster) | PBS | 25.0 (3/12) | 58.3 (7/12) | 16.7 (2/12) | 0 (0/12) | 0.830 |
| | | BTV-4 | 16.7 (2/12) | 50.0 (6/12) | 25.0 (3/12) | 8.3 (1/12) | |
| | | AlOOH | 25.0 (3/12) | 58.3 (7/12) | 8.3 (1/12) | 8.3 (1/12) | |
| | | ApNPs | 16.7 (2/12) | 66.7 (8/12) | 16.7 (2/12) | 0 (0/12) | |
| | | MCT | 9.1 (1/11) | 45.5 (5/11) | 36.4 (4/11) | 9.1 (1/11) | |
| | **Total** | PBS | 16.7 (4/24) | 62.5 (15/24) | 20.8 (5/24) | 0 (0/24) | 0.115 |
| | | BTV-4 | 13.0 (3/23) | 56.5 (13/23) | 21.7 (5/23) | 8.7 (2/23) | |
| | | AlOOH | 13.0 (3/23) | 56.5 (13/23) | 17.4 (4/23) | 13.0 (3/23) | |
| | | ApNPs | 16.7 (4/24) | 75.0 (18/24) | 8.3 (2/24) | 0 (0/24) | |
| | | MCT | 18.2 (4/22) | 31.8 (7/22) | 36.4 (8/22) | 13.6 (3/22) | |

| | | | | | | | |
|---|---|---|---|---|---|---|---|
| MH | Right (prime) | PBS | 0 (0/12) | 33.3 (4/12) | **66.7 (8/12)↑↑↑** | **0 (0/12)↓** | **0.018*** |
| | | BTV-4 | 0 (0/11) | 54.5 (6/11) | 9.1 (1/11) | 36.4 (4/11) | |
| | | AlOOH | 0 (0/11) | 45.5 (5/11) | 27.3 (3/11) | 27.3 (3/11) | |
| | | ApNPs | 0 (0/12) | 66.7 (8/12) | 25.0 (3/12) | 8.3 (1/12) | |
| | | MCT | 0 (6/11) | 54.5 (6/11) | 9.1 (1/11) | 36.4 (4/11) | |
| | Left (booster) | PBS | 0 (0/12) | 33.3 (4/12) | 41.7 (5/12) | 25.0 (3/12) | 0.659 |
| | | BTV-4 | 0 (0/12) | 66.7 (8/2) | 16.7 (2/12) | 16.7 (2/12) | |
| | | AlOOH | 0 (0/12) | 75.0 (9/12) | 16.7 (2/12) | 8.3 (1/12) | |
| | | ApNPs | 0 (0/12) | 50.0 (6/12) | 33.3 (4/12) | 16.7 (2/12) | |
| | | MCT | 0 (0/11) | 63.6 (7/11) | 27.3 (3/11) | 9.1 (1/11) | |
| | **Total** | PBS | 0 (0/24) | 33.3 (8/24) | 54.2 (13/24) | 12.5 (3/24) | 0.120 |
| | | BTV-4 | 0 (0/23) | 60.9 (14/23) | 13.0 (3/23) | 26.1 (6/23) | |
| | | AlOOH | 0 (0/23) | 60.9 (14/23) | 21.7 (5/23) | 17.4 (4/23) | |
| | | ApNPs | 0 (0/24) | 58.3 (14/24) | 29.2 (7/24) | 12.5 (3/24) | |
| | | MCT | 0 (0/22) | 59.1 (13/22) | 18.2 (4/22) | 22.7 (5/22) | |
| FH | Right (prime) | PBS | 8.3 (1/12) | 58.3 (8/12) | 33.3 (3/12) | 0 (0/12) | **0.039*** |
| | | BTV-4 | 18.2 (2/11) | 18.2 (8/11) | 54.5 (1/11) | 9.1 (0/11) | |
| | | AlOOH | 0 (0/11) | **9.1 (3/11)↓** | **45.5 (5/11)↑** | **45.5 (3/11)↑↑↑** | |
| | | ApNPs | 25.0 (3/12) | 66.7 (8/12) | 8.3 (1/12) | 0 (0/12) | |
| | | MCT | 18.2 (2/11) | 63.6 (7/11) | 18.2 (2/11) | 0 (0/11) | |
| | Left (booster) | PBS | 0 (0/12) | 91.7 (11/12) | 8.3 (1/12) | 0 (0/12) | **0.008**** |
| | | BTV-4 | 25.0 (3/12) | 66.7 (8/12) | 8.3 (1/12) | 0 (0/12) | |
| | | AlOOH | 0 (0/12) | 83.3 (10/12) | 16.7 (2/12) | 0 (0/12) | |
| | | ApNPs | **50.0 (6/12)↑↑↑** | **50.0 (6/12)↓** | 0 (0/12) | 0 (0/12) | |
| | | MCT | 9.1 (1/11) | 90.9 (10/11) | 0 (0/11) | 0 (0/11) | |
| | **Total** | PBS | 8 (1/24) | 41.7 (19/24) | 45.8 (4/24) | 4.2 (0/24) | **0.001**** |
| | | BTV-4 | 8.7 (5/23) | 43.5 (16/23) | 39.1 (2/23) | 8.7 (0/23) | |
| | | AlOOH | **0 (0/23)↓** | 21.7 (13/23) | **47.8 (7/23)↑↑** | **30.4 (3/23)↑↑↑** | |
| | | ApNPs | **12.5 (9/24)↑↑↑** | 62.5 (14/24) | 20.8 (1/24) | 4.2 (0/24) | |
| | | MCT | 0 (3/22) | 40.9 (17/22) | 27.3 (2/23) | 31.8 (0/22) | |

*Histol cat.*, histological category of lymphoid hyperplasia; *CPT*, cortex-paracortex thickness; *SF*, secondary follicles; *MP*, medullary plasmacytosis; *MH*, medullary histiocytosis; *FH*, follicular hyalinosis; *LR*, likelihood-ratio; $\chi^2$, chi-square test. [a]Data in red with arrows indicate significance, direction, and magnitude based on adjusted residuals: **p* < 0.05 (one arrow), ***p* < 0.01 (two arrows), ****p* < 0.001 (three arrows). Arrows indicate the direction and magnitude of significance based on analysis of adjusted residuals: one arrow (**p* < 0.05), two arrows (***p* < 0.01), three arrows (****p* < 0.001).

**Table S13.** Comparative analysis of the presence of macrophage aggregates (*M*φ) by group.

| Histol cat. | Side | Group | Grade (%, absolute frequencies)[a] 0 | 1 | 2 | 3 | $p_{LR/\chi^2}$ |
|---|---|---|---|---|---|---|---|
| Aggregates of Mφ | Right (prime) | PBS | 50.0 (6/12) | 50.0 (6/12) | - | - | **<0.001 ***** (LR) |
| | | BTV-4 | 54.5 (6/11) | 45,5 (5/11) | - | - | |
| | | AlOOH | **9.1 (1/11)↓↓↓** | **90.9 (10/11)↑↑↑** | - | - | |
| | | ApNPs | **100.0 (12/12)↑↑** | **0 (0/12)↓↓** | - | - | |
| | | MCT | 81.8 (9/11) | 18.2 (2/11) | - | - | |
| | Left (booster) | PBS | 58.3 (7/12) | 41.7 (5/12) | - | - | 0.101 ($\chi^2$) |
| | | BTV-4 | 58.3 (7/12) | 41.7 (5/12) | - | - | |
| | | AlOOH | 25.0 (3/12) | 75.0 (9/12) | - | - | |
| | | ApNPs | 75.0 (9/12) | 25.0 (3/12) | - | - | |
| | | MCT | 72.7 (8/11) | 27.3 (3/11) | - | - | |
| | Total | PBS | 54.2 (13/24) | 45.8 (11/24) | - | - | **<0.001 ***** ($\chi^2$) |
| | | BTV-4 | 54.2 (13/24) | 45.8 (11/24) | - | - | |
| | | AlOOH | **17.4 (4/23)↓↓↓** | **82.6 (19/23)↑↑↑** | - | - | |
| | | ApNPs | **87.5 (21/24)↑↑↑** | **12.5 (3/24)↓↓↓** | - | - | |
| | | MCT | **77.3 (17/22)↑** | **22.7 (5/22)↓** | - | - | |

*Histol cat.*, histological category evaluated; *M*φ, macrophages; *LR*, likelihood-ratio; $\chi^2$, chi-square test. [a]Data in red with arrows indicate significance, direction, and magnitude based on adjusted residuals: **p* < 0.05 (one arrow), ***p* < 0.01 (two arrows), ****p* < 0.001 (three arrows).

**Table S14.** Adjuvant quantities administered per dose, weight and animal.

| Group | Sheep [Kg] | D ml | Adj C | Total adj/D | Adj/D | Total adj/ animal | Total adj/ animal | P:Adj[a] | |
|---|---|---|---|---|---|---|---|---|---|
| | | | *[mg mL$^{-1}$]* | *[mg]* | *[mg kg$^{-1}$]* | *[mg]* | *[mg kg$^{-1}$]* | *Prime* | *Boost* |
| PBS *n* = 6 | 47.1 ± 10 | | 0 | 0 | 0 | 0 | 0 | - | - |
| BTV-4 *n* = 6 | 48.3 ± 8.3 | | 0 | 0 | 0 | 0 | 0 | - | - |
| AlOOH *n* = 6 | 53.5 ± 6.8 | 2 | 6 ($Al^{+3}$: 2.34 ± 0.00)[a] | 12 ($Al^{+3}$: 4.68) | 0.24 ± 0.03 ($Al^{+3}$: 0.09 ± 0.01) | 24 ($Al^{+3}$: 9.36) | 0.46 ± 0.06 ($Al^{+3}$: 0.18 ± 0.02) | 1:0.24 | 1:0.47 |
| ApNPs *n* = 6 | 48.7 ± 2.1 | | 6.25 | 12.5 | 0.26 ± 0.01 | 25 | 0.51 ± 0.02 | 1:0.24 | 1:0.47 |
| MCT *n* = 6 | 52.8 ± 3.9 | | 20 | 40 | 0.76 ± 0.06 | 80 | 1.52 ± 0.11 | 1:0.78 | 1:1.57 |

*Kg*, kilograms; *D*, dose; *Adj C*, adjuvant concentration; *Adj*, adjuvant; *P:Adj*, ratio of viral protein:adjuvant; [a] A Bradford assay was performed to analyze protein concentration from purified viral solution; [b] $Al^{+3}$ content in triplicate AlOOH samples (6 mg mL$^{-1}$ in PBS) estimated by ICP-MS.

**Table S15.** Microscopic evaluation and score of the lymphoid hyperplasia changes observed in prescapular and axillary lymph nodes.

| Histological change | Description | Score |
|---|---|---|
| Cortex-paracortex thickening (CPT)[a] | Insignificant | 0 |
| | Slight cortex-paracortex area thickening. | 1 |
| | Moderate cortex-paracortex area thickening. | 2 |
| | Marked, diffuse cortex-paracortex area thickening. | 3 |
| Secondary follicles (SF) | Insignificant | 0 |
| | <50% of follicles exhibit germinal centers | 1 |
| | 50-70% of follicles exhibition germinal centers | 2 |
| | >75% of follicles exhibit germinal enters | 3 |
| Medullary plasmacytosis (MP) | Insignificant | 0 |
| | <50% of cells in medullary cords are plasma cells | 1 |
| | 50-70% of cells in medullary cords are plasma cells | 2 |
| | >75% of cells in medullary cords are plasma cells | 3 |
| Medullary sinus histiocytosis (MH) | Insignificant | 0 |
| | <50% of medullary sinuses are occupied by histiocytes | 1 |
| | 50-70% of medullary sinuses are occupied by histiocytes | 2 |
| | >75% of medullary sinuses are occupied by histiocytes | 3 |
| Follicular hyalinosis (FH)[a] | Insignificant | 0 |
| | <50% of follicles exhibit homogenous eosinophilic material | 1 |
| | 50-75% of germinal centers exhibit homogenous eosinophilic material | 2 |
| | >75% of germinal centers exhibit homogenous eosinophilic material | 3 |

[a] Cellularity of cortex and paracortex compartments were jointly assessed.

## 1.3. Supporting Texts

### 1.3.1. Supporting Text 1. Adjuvants and viral characterization

Comprehensive characterization of the materials was performed, including particle size distribution (both hydrodynamic and dry diameters), ζ potential analysis, and structural identification of the three adjuvant and the inactivated BTV-4 solution. Adjuvant identification was conducted using X-ray diffraction with a PANalytical Empyrean system in Bragg-Brentano configuration (Cu Kα radiation; λ=1.54 Å, 45 kV, 40 mA). The diffractogram of microcrystalline tyrosine (MCT) was recorded over a $2\theta$ range from 10º to 50º, with a step size of 0.01º and analysis time of 200 s. The diffractogram for the aluminum oxyhydroxide adjuvant (AlOOH) adjuvant was analyzed from 10º to 80º, while the apatite nanoparticles (ApNPs) diffractogram was analyzed from 15º to 50º, both with a step size of 0.01º and an analysis time of 150 seconds. For comparative analysis with biomimetic ApNPs, a powdered sample of mineral hydroxyapatite (Sigma-Aldrich, UK) and a sample from the diaphysis of a mid-aged sheep femur were included. The fresh bone was treated with hydrogen peroxide (3%) to remove organic matter and subsequently ground into a fine powder. The particle size of ApNPs (0.1 mg $mL^{-1}$) and AlOOH (0.1 mg $mL^{-1}$) samples was determined by transmission electron microscopy (TEM), measuring crystal dimensions (lengths and widths for ApNPs) or microaggregate sizes (for AlOOH). TEM imaging was performed using a T-20 FEI Tecnai microscope equipped with a LaB6 electron source and a SuperTwin® objective, providing a 2.4 Å point-to-point resolution at 200 kV. Samples were pipetted onto 200 Cu formvar carbon TEM grids and air-dried at room temperature for 12 h before imaging. For comparison, the same AlOOH adjuvants (Adjuval® and Alhydrogel®, the standard AlOOH adjuvant), were prepared following the same protocol and analyzed using a high-angle annular dark-field scanning transmission electron microscope (HAADF-STEM) with a Tecnai G2-F30 field emission gun transmission electron microscope (FEG-TEM) (Thermofisher®) operated at 300 kV. In samples of MCT crystals (2 mg $mL^{-1}$) the size was determined using Neubauer counting chamber and optical microscopy. Further morphological characterization of MCT was performed using high-vacuum field emission scanning electron microscopy (SEM) with an Inspect 50 (FEI Company, Hillsboro, USA) operating at 10 kV acceleration voltage. A sample matching the MCT concentration in the vaccine (20 mg $mL^{-1}$, PBS) was air-dried after being placed on a glass slide and secured with carbon tape to an SEM sample holder. Z potential was measured using Phase Analysis Light Scattering (PALS) and analyzed with PALS Zeta Potential Analyzer software (Brookhaven Instruments

Corporation, USA). Ten measurements, with a minimum of 10 cycles per run, were performed on triplicate samples (0.1 mg mL$^{-1}$) of AlOOH, ApNPs and MCT using the Smoluchowski approximation, as well as a solution of inactivated BTV-4 in PBS at the concentration used for booster formulations. Laser diffraction analysis was conducted to assess the volume-based particle size distribution of the aggregates of the adjuvants alone in PBS, as well as those in their respective inocula after incorporation into booster formulations. This analysis was conducted in triplicate using a Mastersizer 3000E (Malvern Instruments Ltd., Malvern, UK) equipped with a HydroSV liquid sample dispersion unit. The volume-weighted size parameters D[4,3], Dv10, Dv50, Dv90 and span were obtained and analyzed using Mie theory and the corresponding respective refractive indices for each material, the were obtained and analyzed.

**1.3.2. Supporting Text 2. Complete cell blood counts and clinical chemistry**

For complete cell blood counts (CBC), the following parameters were determined: erythrocytes (RBC; x$10^6$ μL$^{-1}$), hematocrit (Hct; %), hemoglobin (Hb; g dL$^{-1}$), mean corpuscular volume (MCV; f L$^{-1}$), mean corpuscular hemoglobin (MCH; pg), mean corpuscular hemoglobin concentration (MCHC; g dL$^{-1}$), platelets (PLT; x$10^3$ μL$^{-1}$), monocytes (x$10^3$ μL$^{-1}$), neutrophils (x$10^3$ μL$^{-1}$), lymphocytes (x$10^3$ μL$^{-1}$), eosinophils (x$10^3$ μL$^{-1}$) and basophils (x$10^3$ μL$^{-1}$). For serum biochemistry, the following parameters were measured: blood urea nitrogen (BUN; mg dL$^{-1}$), creatinine (Crea; mg dL$^{-1}$), gamma-glutamyl transferase (GGT; U L$^{-1}$), total bilirubin (TBil; mg dL$^{-1}$), alanine aminotransferase (ALT; U L$^{-1}$), aspartate aminotransferase (AST; U L$^{-1}$), calcium ($Ca^{2+}$; mg dL$^{-1}$) and phosphorus (PHOS; mg dL$^{-1}$).

## 1.4. References of supporting information


(1) Pakharukova, V. P.; Shalygin, A. S.; Gerasimov, E. Y.; Tsybulya, S. V; Martyanov, O. N. Structure and Morphology Evolution of Silica-Modified Pseudoboehmite Aerogels during Heat Treatment. *J. Solid State Chem*. **2016**, *233*, 294–302. https://doi.org/https://doi.org/10.1016/j.jssc.2015.11.007.

(2) Okada, K.; Nagashima, T.; Kameshima, Y.; Yasumori, A.; Tsukada, T. Relationship between Formation Conditions, Properties, and Crystallite Size of Boehmite. *J. Colloid Interface Sci.* **2002**, *253* (2), 308–314. https://doi.org/https://doi.org/10.1006/jcis.2002.8535.

(3) Shardlow, E.; Exley, C. The Size of Micro-Crystalline Tyrosine (MCT®) Influences Its Recognition and Uptake by THP-1 Macrophages: In Vitro. *RSC Adv*. **2019**, *9* (42), 24505–24518. https://doi.org/10.1039/c9ra03831k.

(4) Reece, W. O.; Erickson, H. H.; Goff, J. P.; Uemura, E. E. Dukes' Physiology of Domestic Animals. 13th ed. John Wiley & Sons Inc.: Ames, Iowa 2015, p 768.
